# Isolation, Identification, and Genomic Analysis of a Novel Reovirus from Healthy Grass Carp and Its Dynamic Proliferation In Vitro and In Vivo

**DOI:** 10.3390/v13040690

**Published:** 2021-04-16

**Authors:** Ke Zhang, Wenzhi Liu, Yiqun Li, Yong Zhou, Yan Meng, Lingbing Zeng, Vikram N. Vakharia, Yuding Fan

**Affiliations:** 1 National Demonstration Center for Experimental Fisheries Science Education, Shanghai Ocean University, Shanghai 201306, China; zhangke7201@163.com; 2 Yangtze River Fisheries Research Institute, Chinese Academy of Fishery Sciences, Wuhan 430223, China; liuwenzhialisa@yfi.ac.cn (W.L.); liyq@yfi.ac.cn (Y.L.); zhouy@yfi.ac.cn (Y.Z.); mengy@yfi.ac.cn (Y.M.); zlb@yfi.ac.cn (L.Z.); 3 Institute of Marine and Environmental Technology, University of Maryland Baltimore County, Baltimore, MD 21202, USA; vakharia@umbc.edu

**Keywords:** healthy grass carp reovirus (HGCRV), grass carp reovirus (GCRV), genome, phylogenetic relationship, proliferate

## Abstract

A new grass carp reovirus (GCRV), healthy grass carp reovirus (HGCRV), was isolated from grass carp in 2019. Its complete genome sequence was determined and contained 11 dsRNAs with a total size of 23,688 bp and 57.2 mol% G+C content, encoding 12 proteins. All segments had conserved 5' and 3' termini. Sequence comparisons showed that HGCRV was closely related to GCRV-873 (GCRV-I; 69.57–96.71% protein sequence identity) but shared only 22.65–45.85% and 23.37–43.39% identities with GCRV-HZ08 and Hubei grass carp disease reovirus (HGDRV), respectively. RNA-dependent RNA-polymerase (RdRp) protein-based phylogenetic analysis showed that HGCRV clustered with *Aquareovirus*-C (*AqRV*-C) prior to joining a branch common with other aquareoviruses. Further analysis using VP6 amino acid sequences from Chinese GCRV strains showed that HGCRV was in the same evolutionary cluster as GCRV-I. Thus, HGCRV could be a new GCRV isolate of GCRV-I but is distantly related to other known GCRVs. Grass carp infected with HGCRV did not exhibit signs of hemorrhage. Interestingly, the isolate induced a typical cytopathic effect in fish cell lines, such as infected cell shrank, apoptosis, and plague-like syncytia. Further analysis showed that HGCRV could proliferate in grass carp liver (L28824), gibel carp brain (GiCB), and other fish cell lines, reaching a titer of up to 7.5 × 10^4^ copies/μL.

## 1. Introduction

Grass carp (*Ctenopharyngodon idella*) is an important freshwater aquaculture fish that is cultured widely in Asian countries. However, serious outbreaks of grass carp hemorrhagic disease (GCHD) occur frequently in many freshwater farms in southern China, which is associated with high mortality (up to 85% of fingerlings and yearlings), causing great economic losses [1,2]. The pathogen that causes GCHD was named grass carp reovirus (GCRV) and was identified as a new member of the family *Reoviridae* in 1983 [3]. The genus *Aquareovirus* was initially classified in the family *Reoviridae* in 1991 [4]. To the best of our knowledge, there is no standard criterion for the species classification of the *Aquareovirus*; seven species (*AqRV*-A to *AqRV*-G) and some other unassigned species have been recognized, mainly based on the RNA-RNA hybridization analyses, sequence analysis, antigenic properties, and other characteristics. Recent studies have found that the identification of species belonging to the *Aquareovirus* genus has been mainly based on the RNA-dependent RNA-polymerase protein sequence (RdRp) [5,6,7,8,9,10,11]. When the RdRp amino acid sequence has <74% identity, species segregation is recommended, whereas sequence identity >95% warrants species integration [12]. However, if the sequence identity is between 74 and 95%, there is considerable uncertainty and variability for species classification [12]. Some additional factors should be considered in these instances, such as genome segments, host range, disease symptoms, electropherotype analysis, serological comparison, and ability to reassort during mixed infections.

Until now, different GCRV strains have been isolated from diseased grass carps with typical signs of hemorrhage [13,14,15,16,17,18,19,20,21,22,23,24,25]. Meanwhile, according to the genome sequence analyses and biological characteristics, GCRV isolates in China are mainly divided into three genotypic groups, namely GCRV-I (representative strain GCRV-873; *Aquareovirus*-C), GCRV-II (representative strain GCRV-HZ08), and GCRV-III (representative strain HGDRV).

A number of GCRV isolates have been isolated, and their genomes have been completely sequenced [8,9,10,13,14,23], including GCRV-873, GCRV-GZ1208, GCRV-AH528, GCRV-HZ08, GCRV-GD108, GCRV-106, GCReV-109, GCRV-918, HGDRV (GCRV-104), GCRV-Hunan794, GCRV-Huan1307, and GCRV-Henan988, which comprises 11 segments of double-stranded RNA (S1–S11). Meanwhile, GCRV strains are heterogenous, and different isolates have marked differences in virulence, cell culture characteristics, immunogenicity, and pathogenicity to grass carp [1,14,16,17,20]. For example, the virulence of genotypes I and III is weak [1,20]. In the present study, a new strain of reovirus strain was found in grass carp during a routine examination in Hubei province, China, in 2019. Given that the grass carp were healthy, we hypothesized that the carp might be carriers, the virus is nonpathogenic, or it has low virulence in grass carp. In this study, we identified this new reovirus strain as healthy grass carp reovirus (HGCRV), and its biological characteristics were studied. Moreover, the complete genomic sequence of HGCRV was determined and analyzed, which exhibited distinct molecular characteristics. In addition, the in vitro and in vivo proliferation of this virus was detected. This study provides a deeper understanding of the molecular epidemiology and genetic diversity of GCRV strains in China.

## 2. Materials and Methods

### 2.1. Virus Isolation and Culture

Grass carp tissues were collected from healthy grass carp in Hubei province. Firstly, liver, kidney, and spleen tissues were cut into small pieces and homogenized in phosphate-buffered saline (PBS). The homogenate was centrifuged at 4 °C at 2880× *g* for 30 min after freeze-thawing at −80 °C three times. Then, the supernatant was filtered and sterilized through a 0.22 μm microporous membrane and stored at −80 °C for further use. The grass carp ovary (GCO) cell line was kindly provided by Dr. Qiya Zhang (Institute of Hydrobiology, Chinese Academy of Sciences) and cultured in T-25 cm2 cell culture flasks with M199 medium containing 10% (*v*/*v*) fetal bovine serum (FBS) (Every Green, Zhejiang, China) at 25 °C. After removing the culture medium from the 85% confluent cell monolayer, 1 mL of virus suspension was inoculated onto the cells and incubated for 1 h at 25 °C. Then, the supernatant was removed, and 5 mL of M199 with 2% FBS was added. The infected cells were further cultured at 25 °C for 6 days, and the cytopathic effect (CPE) was observed daily. Non-infected cells were used as a control. The whole-cell culture medium was collected after freeze-thawing at −80 °C three times, followed by centrifugation at 2880× *g* for 30 min to remove cell debris. The supernatant was collected as a viral suspension and stored for further use.

### 2.2. Artificial Infection

Healthy grass carp fingerlings, with a body length of 5–10 cm and an average weight of 4 ± 1.5 g, were acclimatized to laboratory conditions for two weeks (50 fish in each tank) with aerated water at 28 °C before the experimental challenge. We randomly sampled for detection of GCRV by RT-PCR to ensure that these fish were free of this virus. Then the test group was artificially infected by intraperitoneal injection with 0.1 mL of the sixth passage of HGCRV, and the control group was intraperitoneally injected with 0.1 mL of PBS. The virus was re-isolated from the experimentally infected fish, and the propagated virus was used to infect the healthy fish again, using the method described above. Finally, fish were collected from each test and were anesthetized by immersion in MS222 solution (100 mg/L). Then, tissue samples were collected on the ice.

### 2.3. Physical and Chemical Properties

Chloroform and diethyl ether sensitivity was determined as described previously [10]. Acid-base sensitivity was tested by keeping an aliquot of the virus at pH 3.0 in 0.1 M HCl and 5.6% NaHCO_3_ for 1 h at 4 °C, which was then regulated to pH 7.2 using 5.6% NaHCO_3_ and 0.1 M HCl [25]. Heat stability was tested by incubating the viral suspension at 56 °C for 30 min and 60 min. Virus titers were determined and calculated by Reed and Muench (1938).

### 2.4. Electron Microscopy and SDS-PAGE

On day 5, virus-infected cells with a CPE were harvested and fixed with glutaraldehyde and osmium acid fixator, dehydrated in an ethanol gradient, and embedded with embedding agent to make ultrathin sections. The sections were examined using transmission electron microscopy (Hitachi-7650, Tokyo, Japan).

The viral supernatant was subjected to ultracentrifugation at 112,700× *g* (Beckman Optima L-80XP Rotor SW28, Beckman Coulter, Indianapolis, IN, USA) for 2 h at 4 °C. The pellet was resuspended in 1 mL distilled deionized water. Total RNA was extracted from the purified viral pellet using TRIzol reagent (Invitrogen, Waltham, MA, USA), according to the manufacturer’s protocol. The extracted RNA was stored at −80 °C. Viral dsRNA were separated by SDS-PAGE on vertical slab gels (4% polyacrylamide gel, 60 V, 10 h) in Laemmli’s buffer (Laemmli, 1970) and then visualized using silver staining. The GCRV standard strains (GCRV-0901, GCRV-HZ08, and HGDRV) were used as controls in SDS-PAGE.

### 2.5. Full-Length Amplification, Cloning, and Nucleotide Sequencing

HGCRV genome sequences were obtained by Illumina sequencing (Illumina, San Diego, CA, USA). However, multiplex Illumina sequencing was not capable of obtaining the entire HGCRV genome without pre-amplification of the sample. For this isolate, only the full-length sequences of segments S10 and S11 were obtained by the Illumina sequencing. Therefore, we designed primers and amplified the entire HGCRV genome using SMARTer RACE 5′/3′ Kit (Clontech, Mountain View, CA, USA). The primers and their sequences are listed in Table 1. The reaction program was 98 °C 10 s, 65 °C 10 s, 72 °C 1 min, 30 cycles. Then PCR products were purified using a gel extraction kit (Omega, Winooski, VT, USA) according to the manufacturer’s instruction and cloned into a pEASY^®^-Blunt Zero Cloning vector (TransGen Biotech, Beijing, China). The ligation product was transfected into Trans1-T1 phage-resistant chemically competent cells. Positive recombined plasmids were sequenced using M13 forward and M13 reverse primers. The whole-genome sequence was obtained by a splicing method using SEQMAN software (Lasergene, DNASTAR Inc., Madison, WI, USA). The genomic DNA sequences and deduced amino acid sequences of HGCRV were provided in the Supplementary Materials (S1) .

### 2.6. Sequence Analysis

The *Aquareovirus* and other fish reovirus strain sequences used for comparisons in this study were obtained from GenBank, and BLAST searches were used to find related nucleic acid and protein entries in the database at the National Centre for Biotechnology Information server (NCBI). Genomic DNA sequences and deduced amino acid sequences of HGCRV were analyzed using EditSeq (DNASTAR 5.0). In addition, hydrophobicity and conserved domains within the proteins were predicted using the ExPASy server (SIB Swiss Institute of Bioinformatics, Lausanne, Switzerland) and the Simple Modular Architecture Research Tool (SMART). Multiple sequence alignments were performed using the ClustalW software program and Molecular Evolutionary Genetics Analysis version 7.0 (MEGA7.0). Phylogenetic trees were constructed using the neighbor-joining method of the mega program based on RdRp and VP6 sequences.

### 2.7. HGCRV Proliferation In Vitro and In Vivo

Healthy grass carp were placed in different tanks and infected. The infected and control groups were tested in triplicate and 50 fish in each group. Then the liver, kidney, and spleen tissues were harvested in triplicate each day. For in vitro culture, *Ctenopharyngodon idelus* kidney (CIK), gibel carp brain (GiCB), and *Epithelioma papulosum* cyprinid (EPC) cell lines were maintained in our laboratory, and these cell lines were used in the present study. The grass carp liver (L8824) cell line was purchased from Wuhan University, China Center for Type Culture Collection. These cell lines were infected with the HGCRV, and the state of the cells was examined microscopically daily. Cells were harvested for the triplicate test at different times. Total RNA was extracted from these tissues and cell samples using the TRIzol reagent (Invitrogen, Waltham, MA, USA). cDNA was synthesized using an EasyScript^®^ One-Step gDNA Removal and cDNA Synthesis SuperMix kit (TransGen Biotech), according to the manufacturer’s protocol. The standard curve was obtained by constructing the nearly full-length vector of S10. Then triplicate quantitative real-time fluorescence PCR (qPCR) with primer pairs of S10-F: 5′−CACTGCCAGGCCGGTCAC−3′ and S10-R: 5′−GGTGTCGTGGGCTAGAAGCAG−3′ was used to analyze the proliferation of HGCRV in various cell lines and grass carp tissues using TB Green^®^ Premix Ex Taq™ II (Takara, Dalian, China).

### 2.8. Statistical Analysis

The standard curve was constructed using Excel (Microsoft, Redmond, WA, USA). Then the mean, SEM, and Student’s t-test were conducted using GraphPad Prism version 8.0 (GraphPad Inc. La Jolla, CA, USA). The results are presented as the mean ± SEM. A value of *p* < 0.05 was considered significant. Two-way analysis of variance (ANOVA) was used to analyze viral replication in cells and fish infected with HGCRV, respectively.

## 3. Results

### 3.1. Pathology and Morphology of HGCRV

**Figure 1:**
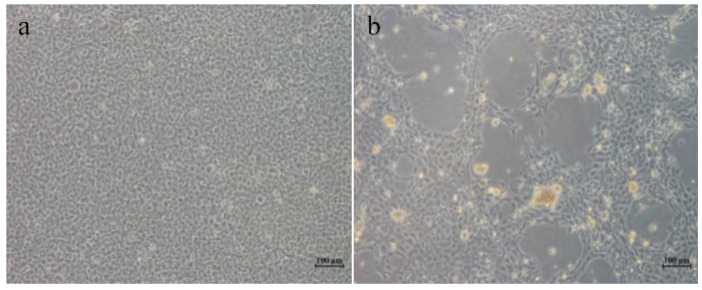
Cytopathic effect (CPE) induced by healthy grass carp reovirus (HGCRV) in a grass carp ovary (GCO) cell line. (**a**) Uninfected GCO cells. (**b**) CPE in GCO cells at 72 h after infection. CPE, cytopathic effect; HGCRV, healthy grass carp reovirus; GCO, grass carp ovary.

HGCRV was cultured in a GCO cell line from homogenates of grass carp tissues at 25 °C. It could induce plaque-like syncytia as the typical CPE at 5 days after inoculation. After three passages, CPE was produced at 2 days post-infection (Figure 1). Healthy grass carp fingerlings infected with HGCRV did not exhibit signs of hemorrhage, which suggested that the virus might be nonpathogenic or has low virulence in grass carp.

The physical and chemical properties of HGCRV showed some stability under acidic and basic conditions (pH 3 and pH 11). Moreover, HGCRV remained infectious after heating at 56 °C for 30 min and 60 min. Treatment with ether or chloroform did not affect viral infectivity. These properties are similar to those reported for similar viruses in previous studies [25].

**Figure 2:**
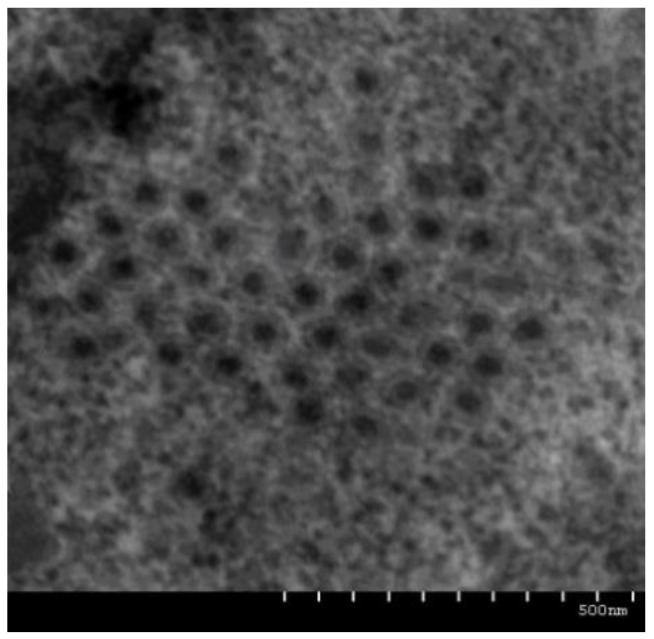
Electron micrograph of HGCRV. GCO cells infected with HGCRV were made into ultrathin slices and observed using transmission electron microscopy. HGCRV, healthy grass carp reovirus; GCO, grass carp ovary.

Transmission electron microscopy of virus-infected GCO cells showed that there were a large number of aggregated and clustered virus particles in the GCO cells, with a diameter of 50–70 nm (Figure 2).

### 3.2. HGCRV Genome

**Figure 3:**
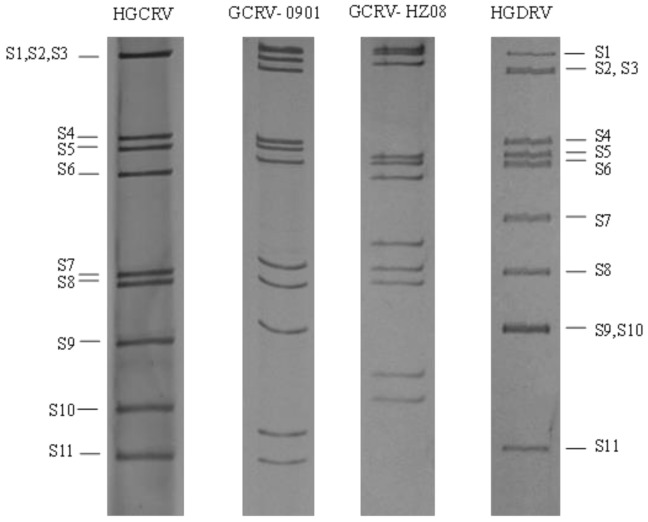
Electropherotype of the HGCRV genome. HGCRV, healthy grass carp reovirus.

The genomes of HGCRV and GCRV representative strains were analyzed by SDS-PAGE of their total RNA. As shown in Figure 3, the HGCRV genome segments separated into nine distinct bands. The high molecular weight segments (S1 to S3) commigrated into one band, and the other eight segments (S4 to S11) migrated individually. Comparison with the genomes of different GCRV representative strains (GCRV-0901/HZ08/HGDRV) showed that the banding pattern of HGCRV genome segments was different from those of GCRV, which suggested that HGCRV is a new strain of GCRV.

The HGCRV genomic RNA contained 11 segments (S1−S11). The length of individual genome segments, the proteins they encode, the predicted functions of the encoded proteins, and the conserved terminal sequences of each segment are listed in Table 2. The segment size ranged from 821 to 3949 bp, with a total size of 23,688 bp. The G+C content was determined as 57.2 mol%, which was similar to previously sequenced aquareoviruses (52–60%). Each genome segment encoded one protein, except for the S7 segment, which encoded two proteins. The 12 proteins had predicted sizes ranging from 35.5 to 141.26 kDa (Table 2).

Short non-coding regions (NCRs) were found at the 5’ and 3’ terminal regions in all of the HGCRV genome segments, which are characteristic features of aquareovirus genomes. The length of HGCRV NCRs ranged from 12 to 43 nucleotides at the 5’ end and 35 to 70 nucleotides at the 3’ end. Analysis of the 5’ and 3’ NCRs showed that all gene fragments shared a 5’−GUUAUU motif in the 5’ NCRs and a UCAUC−3’ conserved motif in the 3’ NCRs (Table 2).

### 3.3. Sequence Analysis of the HGCRV

The S1 genome segment of HGCRV was predicted to encode the core protein VP1 (1299 amino acids (AA)). BLASTp searches showed that VP1 possessed putative conserved domains that belonged to the reovirus L2 superfamily, consisting of several reovirus core-spike protein lambda-2 (L2) sequences, indicating that VP1 functions as the mRNA capping enzyme [26,27]. The VP1 protein of HGCRV shared sequence identities between 29.48 and 90.76% with the VP1 protein of related aquareoviruses, including CHSRV (*AqRV*-A), SMReV (*AqRV*-A), FCAV (*AqRV*-B), GCRV873 (*AqRV*-C), GSRV (*AqRV*-C), AGCRV (*AqRV*-G), GCRV-HZ08 (unclassified), GCRV-GD108 (unclassified), and HGDRV (unclassified).

The S2 genome segment was predicted to encode the core protein VP2 (1274 AA), which is an RNA-dependent RNA-polymerase (RdRp). The catalytic domain of RdRp was identified between amino acids 561 and 798 in HGCRV VP2 using ScanProsite. HGCRV VP2 possesses 43.39–94.34% identity with VP2 homologs from related aquareoviruses.

The S3 genome segment encodes the core protein VP3 (1214 AA), which functions as an NTPase and helicase. A zinc finger domain (Cys-His-Cys-His) was identified in the HGCRV VP3 at amino acid positions 117–140, which is known to bind RNA. Four consecutive residues Glu502, Ser503, Thr504, and Thr505 of VP3 are conserved among GCRV and orthoreovirus and are involved in RNA transcription in GCRV [26]. The corresponding amino acids in HGCRV were consistent with previous studies. HGCRV VP3 shares 33.87–97.03% identity with homologs of other aquareoviruses.

The S4 genome segment was predicted to encode the nonstructural protein NS79 (739 AA). This segment was smaller than its homologs in other *Aquareovirus* species. In particular, it has been identified that two coiled regions of the C-terminus of NS79 homologous proteins from the different *Aquareovirus* species are important in viroplasmic inclusion bodies (VIB) formation [28]. In HGCRV, two coiled coils were found at amino acid residue positions 510–535 and 700–762 using the coils program. The NS79 protein of HGCRV shared 22.52–79% sequence identity with the NS1 protein of related aquareoviruses. NS79 has a high sequence identity to NS80 (GCRV-I), suggesting that it might possess a similar function to NS80.

The S5 genome segment was predicted to encode the core protein VP5 (728 AA). BLASTp analysis showed that it possesses a conserved domain belonging to the reovirus_Mu2 superfamily. Mu-2 is a microtubule-associated protein and is thought to play a key role in the formation and structural organization of reovirus inclusion bodies [29,30]. The VP5 protein possesses 23.37–85.99% identity with VP5 homologs from related aquareoviruses.

The S6 genome segment was predicted to encode the outer capsid protein VP4 (648 AA). BLASTp analysis of VP4 showed that it possesses a conserved domain belonging to the reovirus_M2 superfamily. Studies have shown that this protein family affects host cell membrane penetration [31]. The same proteolytic cleavage site was predicted in HGCRV VP4 (Asn42 and Pro43) as that in GCRV and other aquareoviruses [32]. In addition, the first three amino acid residues MGN at the N-terminus of VP4 are highly conserved in aquareoviruses [10]. HGCRV VP4 shares 27.33–91.05% identity with VP4 homologs from other aquareoviruses.

The S7 genome segment was predicted to encode NS32 and NS17, the open reading frames of which are separated by 36 bp non-coding regions. There was a transmembrane helix region (^310^ALGLGCIACGIIGVIVVASGLCC^332^), as detected using the TMHMM v2.0 program in NS17. These two proteins share 24.91–72.53% and 30.51–71.92% identity with their homologs from related aquareoviruses, respectively.

The S8 genome segment was predicted to encode the core protein VP6 (412 AA). BLASTp analysis showed that VP6 possesses conserved domains belonging to the reoviral sigma 1/sigma 2 superfamily. Previous studies showed that sigma 1 is a trimeric protein that resides in the outer capsid, and interactions between sigma 2 and lambda 1 and lambda 3 are thought to initiate core formation [33,34]. Studies also suggest that sigma 1 also acts as a cell attachment protein, and determines viral virulence, pathways of spread, tropism, and binds to the lambda 2 core protein [35,36]. VP6 shares 24.92–90.75% identity with its homologs from related aquareoviruses.

The S9 genome segment was predicted to encode the nonstructural protein NS38 (352 AA). BLASTp analysis showed that NS38 possesses a conserved domain belonging to the polyG_pol superfamily. NS3 possesses 37.57–92.05% identity with its homologs from related aquareoviruses.

The S10 genome segment was predicted to encode the outer capsid protein VP7 (276 AA). BLASTp analysis showed that VP7 possesses a conserved domain belonging to the capsid_VP7 superfamily, which is a major viral outermost capsid protein. It might be related to virus entry and interaction between virus and host cells during infection [37]. Sequence alignment shows that HGCRV VP7 has 28.40–70.29% identity to its aquareovirus homologs.

The S11 genome segment was predicted to encode the nonstructural protein NS27 (244 AA). Previous research showed that NS26 can enhance the fusogenic activity of the fusion-associated small transmembrane FAST protein NS16 from *AqRV* [38]. In addition, the ‘TLPK‘ motif is important for the enhancement, especially the lysine (K) residue [39], which existed in the NS27. NS27 protein shares 27.18–77.46% identity with homologous nonstructural aquareovirus proteins.

**Figure 4:**
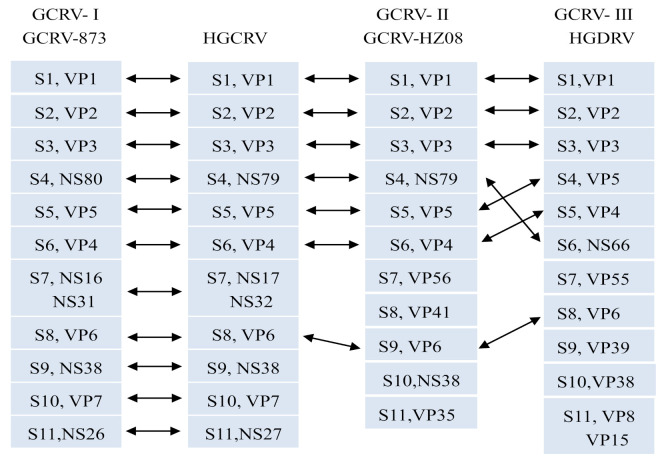
Predicted equivalent relationships of genome segments and proteins between HGCRV, GCRV-873 (GCRV-I), GCRV-HZ08 (GCRV-II), and HGDRV (GCRV-III). Double-headed arrows indicate equivalent segments and proteins. HGCRV, healthy grass carp reovirus.

Taken together, HGCRV encodes 12 proteins, which is consistent with other GCRV strains. According to the above comparison and analysis, the relationship of genome segments and their encoded proteins were similar in HGCRV, GCRV-873, GCRV-HZ08, and HGDRV (Figure 4). The nucleotide and amino acid sequence identity between HGCRV and other GCRV strains are shown in Table 3. Among them, the GCRV-I strains were found to have the highest amino acid sequence identities (69.57–97.03%) compared with other GCRV strains. However, the proteins encoded by S4, S7, S10, and S11 have low amino acid sequence identity with GCRV-I (GCRV-873, 69.57–79%), but they were still higher than those between HGCRV GCRV-II (GCRV-HZ08, 22.65–45.85%) and GCRV-III (HGDRV, 23.37–43.39%).

### 3.4. Phylogenetic Analysis

**Figure 5:**
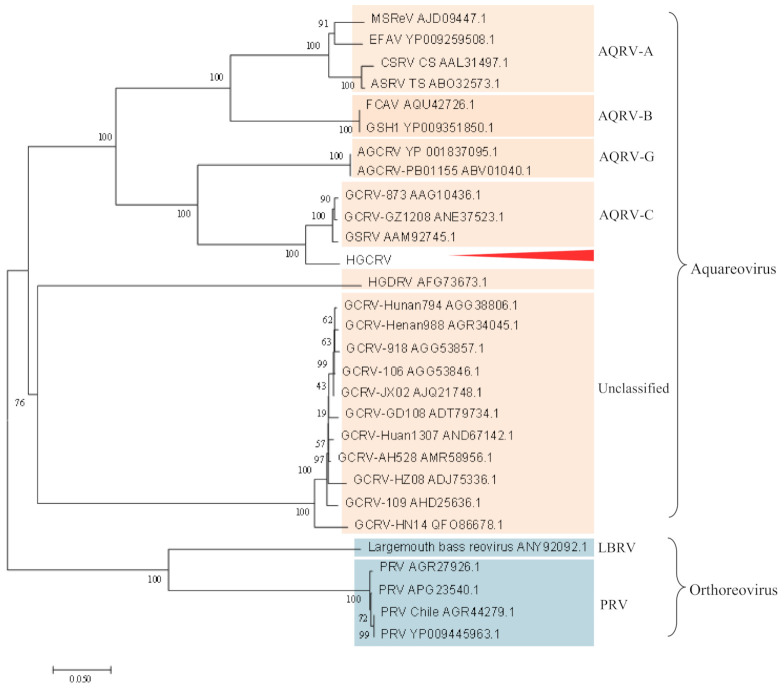
Phylogenetic analysis based on the amino acid sequences of VP2 (RNA-dependent RNA-polymerase (RdRp)) from the genus *Aquareovirus* and fish reovirus. The phylogenetic tree was constructed based on the RdRp proteins of 29 representative strains using the neighbor-joining method with 500 bootstrap replicates. HGCRV is indicated with an arrow. The scale bar indicates the evolutionary distances (the average number of amino acid substitutions per site). RdRp, RNA-dependent RNA-polymerase; HGCRV, healthy grass carp reovirus.

**Figure 6:**
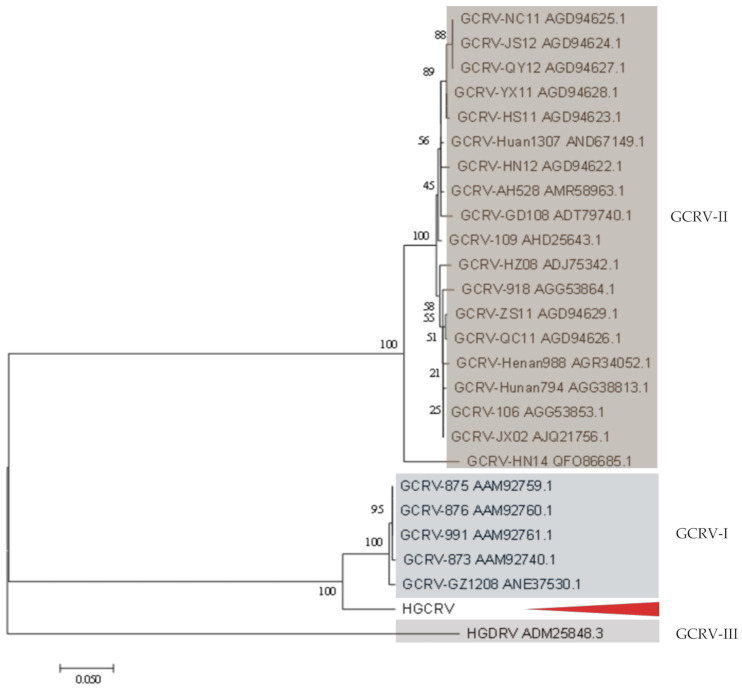
Phylogenetic analysis of HGCRV and other grass carp reovirus strains based on their VP6 (core protein) protein sequences. The analysis involved 26 amino acid sequences, and HGCRV is indicated with an arrow. The phylogenetic tree was constructed using the neighbor-joining method using 500 bootstrap replicates. The scale bar indicates the evolutionary distance (the average number of amino acid substitutions per site). HGCRV, healthy grass carp reovirus.

Studies have shown that the RNA polymerase is a highly conserved protein in the family *Reoviridae* [5,6,10]; therefore, the RdRp protein was used for phylogenetic analysis in this study. A phylogenetic tree was constructed using the neighbor-joining method based on the RdRp proteins of 29 representative strains from aquareoviruses and other fish reoviruses (Figure 5). The result showed that the known species of aquareoviruses clustered in a branch. HGCRV was clustered more closely with *AqRV*-C and tended to cluster with *AqRV*-C before grouping with *AqRV*-G, suggesting that HGCRV might belong to the genus *AqRV*-C but was distinct from the known species. VP6 gene sequences are available for most of the grass carp reovirus strains isolated in China. A phylogenetic tree constructed based on the amino acid sequences of the VP6 protein is shown in Figure 6. This tree showed that grass carp reovirus strains clustered into three genotypic groups. HGCRV is closely related to GCRV-GZ1208, GCRV-873, and other isolated strains in the group GCRV-I, suggesting that it may belong to GCRV-I.

### 3.5. In Vitro and In Vivo Proliferation Characteristics

**Figure 7:**
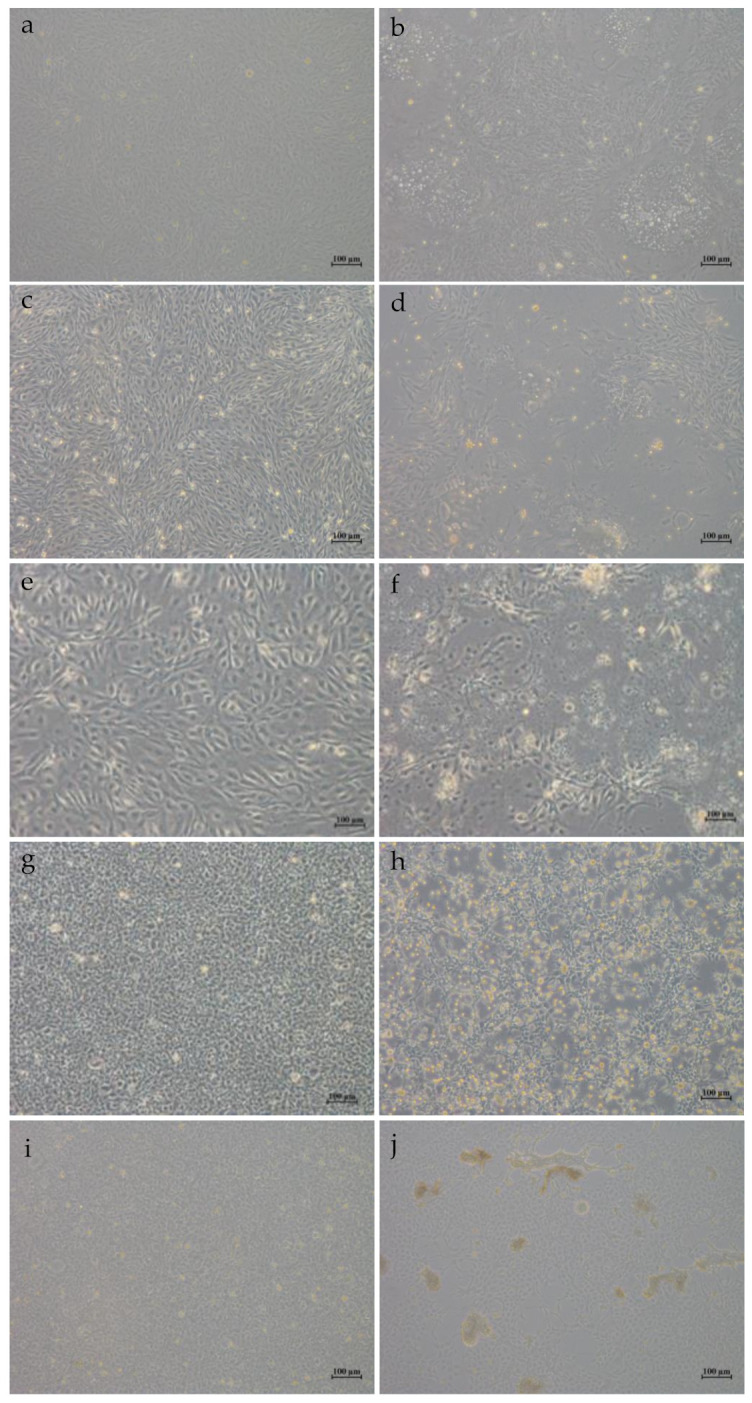
Cytopathic effect in different cell lines after 48 h of virus infection. (**a**,**c**,**e**,**g**,**i**): *Ctenopharyngodon idelus* kidney (CIK), grass carp liver (L8824), gibel carp brain (GiCB), *Epithelioma papulosum* cyprinid (EPC), grass carp ovary (GCO) cells uninfected with HGCRV, respectively; (**b**,**d**,**f**,**h**,**j**): CIK, L8824, GiCB, EPC, GCO cells infected with HGCRV, respectively.

**Figure 8:**
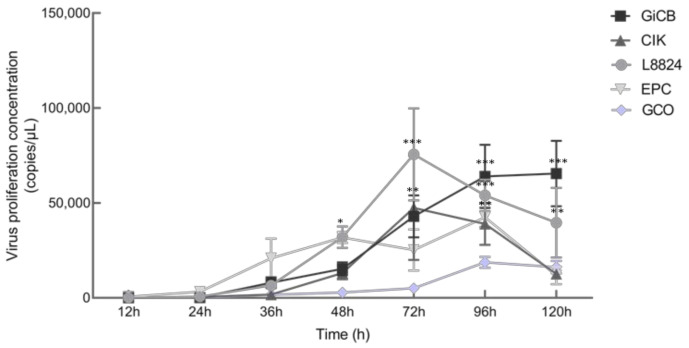
The changes in RNA replication levels in different cell lines infected with HGCRV. HGCRV, healthy grass carp reovirus. * *p* < 0.05, ** *p* < 0.01, *** *p* < 0.001.

**Figure 9:**
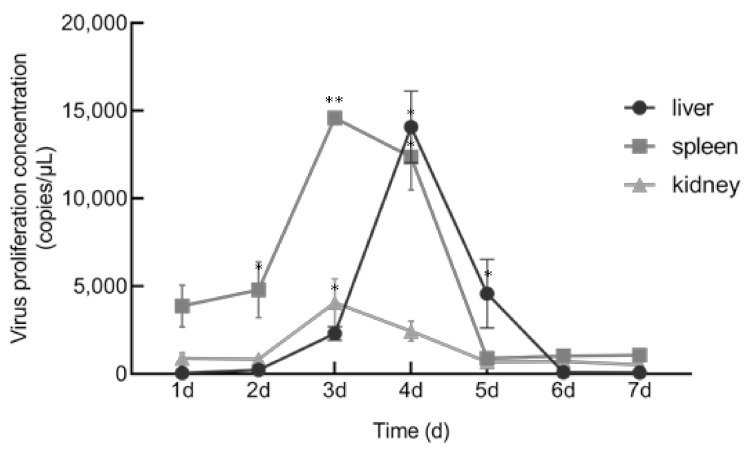
The changes of viral RNA replication levels in different tissues of HGCRV-infected grass carp. HGCRV, healthy grass carp reovirus. * *p* < 0.05, ** *p* < 0.01.

When HGCRV was propagated in vitro, it caused a CPE in CIK, L8824, EPC, and GiCB cell lines (Figure 7). qPCR analysis showed that the HGCRV can proliferate in GCO, CIK, L8824, EPC, and GiCB cell lines (Figure 8), especially in L8824 and GiCB cell lines, in which the virus copies reached 7.5 × 10^4^ and 6.4 × 10^4^ copies/μL, respectively. Interestingly, virus proliferation reached its highest level in L8824, CIK, and EPC cell lines at 72 h, while in GiCB and GCO cells, proliferation peaked at 96 h; however, in all cells, the virus began to proliferate at 24 h. In grass carp, HGCRV can proliferate in the liver, kidney, and spleen tissues, in which tissues the content of viral RNA increased at first and then decreased (Figure 9). The viral titer peaked in the spleen and kidney on the third day after infection, while in the liver, it peaked on the fourth day after infection.

## 4. Discussion

In this study, we reported a new grass carp reovirus, HGCRV. We demonstrated that the virus caused no signs when used to infect healthy grass carp and the passaged virus caused a typical CPE in different cell lines. Previous research showed that although some of the *AqRV*s reported have been isolated during routine examination of apparently healthy fish with no clinical signs. Then, further studies indicated that these could cause subclinical infections and cause significant clinical signs and even severe disease [1,6,40,41,42]. For example, Gao isolated a strain from the healthy grass carp, but it cannot cause CPE in cells and can cause 10% death of the rare minnow (*Gobiocypris rarus*) [1]. However, these characteristics of the HGCRV-infected caused no signs and caused CPE in cells, which is similar to GCRV-0901 and GCRV-873 [20]. However, GCRV-0901 and GCRV-873 were isolated from a diseased grass carp, while HGCRV was found in grass carp samples during the routine examination from Hubei. We hypothesized that it might have evolved from GCRV strains by variations in virulence genes.

Electrophoretic band pattern analysis is an important technique to distinguish the genotypes of segmental RNA viruses [13], which indicated that the HGCRV strain might belong to the GCRV genotype. In general, GCRV genome segments are separated into 11 distinct bands; however, the HGCRV isolate genome only had nine distant bands. However, the control GCRV-0901, GCRV-HZ08, and HGDRV were separated into 11 distinct bands (Figure 3). When we reduced the gel concentration and prolonged electrophoresis time, the large segment S1−S3 was separated into two bands (data not shown). Sequence analysis showed that HGCRV has 11 segments. Therefore, the mobility of the dsRNA segments of GCRV in SDS-PAGE gels depends not only on their size but also on their GC content and secondary structure. Meanwhile, we found that the distribution of the segments closely resembled those of GCRV-0901, but with some differences. These results showed that the electrophoretic band patterns of different isolates can vary, which might be caused by differences in the GC content, the nucleotide sequence, and the number of nucleotides.

Here we further report the complete genome sequence of HGCRV and its deduced protein sequences which share more than 70% identity with the homologous proteins of GCRV-I strains. However, except for S1, S5, S6, and S7, the lengths of the genome sequence fragments were different. Except for the S4 ORF region, the differences were mainly in the non-coding regions. The nucleotide sequences of the segments showed 71.04–79.77% identity to the same fragments of GCRV-I strains (Table 3). In contrast, the lengths of the genome sequences of the GCRV-II and GCRV-III strains are not the same. For the amino acid sequences, the putative protein sequences of S4, S7, S10, and S11 showed lower homology than the other proteins (Table 3). RdRp from isolates of the same species should have an amino acid identity >95%, while the identity between species should be in the range from 57 to 74% [12]. The amino acid identity of HGCRV RdRp was over 74%, but slightly lower than 95% compared with that of *AqRV*-C. Phylogenetic analysis based on the RdRp amino acid sequences also placed HGCRV in a position close to species *AqRV*-C (Figure 5), suggesting that HGCRV could belong to the *AqRV*-C. To analyze the evolutionary relationships among different GCRV isolates, it is necessary to perform genotyping. In GCRV, the VP4, VP6, and VP7 genes encode major outer capsid proteins that are relatively conserved [18]. Therefore, the VP4, VP6, and VP7 genes could be used for GCRV genotyping. Based on VP4, the amino acid identities ranged from 29.4 (HGDRV, GCRV-III) to 91.05% (GCRV-GZ1208, GCRV-I). Phylogenetic analysis based on VP6 placed HGCRV as a sister group to GCRV-I. The amino acid identities ranged from 22.86 (GCRV-HZ08, GCRV-II) to 91.02% (GCRV-GZ1208, GCRV-I). The VP4 and VP6 identities possess > 90% identity with GCRV-I. However, it is interesting that the VP7 protein of HGCRV encoded by S10 shares approximately 70% identity with that of GCRV-873. Previous studies showed that VP7 is relatively conserved within a single species (>80%) [5]; however, lower identity has been observed in different species. Therefore, we believe that the HGCRV isolate might be the same species as GCRV-I but with significant variation.

Fish cell lines can be sensitive to several viruses. Conversely, it is common for a virus to infect several cell lines, and GCRV is less host-specific toward adult fish at the cellular level [43]. Li indicated that GCRV-HZ08 could proliferate in GSB, L8824, PSF, CF, CO, CIB, and KS cell lines [44]. However, the CPE formed in the EPC cell line was not found in GCRV-873 [45], which indicated that the strain is new and is different from other GCRV strains with respect to some properties. For cell culture, viral RNA levels were upregulated rapidly and showed a logarithmic increase from 24 h to peak time. Yuan and Zeng studied the propagation of GCRV-873 and GCRV-854 strains on CIK cells, respectively [46,47]. Their dynamic curves were similar to those in the present study; however, the time of rapid proliferation was different, indicating that different strains had different abilities to infect cells. The viral RNA copies decreased in L8824 and CIK cell lines after 72 h, probably because of apoptosis. Meanwhile, the virus proliferation in fish was detected. These results showed that the viral RNA levels in the liver, spleen, and kidney increased for a short time and then decreased, which might explain the lack of signs and mortality of the infected fish. Wang found that viral RNA levels in the spleen and kidney during GCRV-HZ08 strain infection increased at first and then decreased, which was consistent with the results of the present study [25]. Yuan also observed that RNA expression in liver, kidney, and spleen tissues increased at first and then decreased, with the peak appearing on the second and third days after infection [46]. However, the peak RNA expression in fish tissues appeared on the third and fourth days after HGCRV infection, which indicated that different strains differ in their ability to infect fish. Therefore, we hypothesized that grass carps tested in Hubei are carriers of HGCRV, which is non-virulent, and therefore, further studies are needed to study the molecular basis of its virulence.

## 5. Conclusions

In summary, the present study provided the complete genome sequence of a newly isolated grass carp reovirus from Hubei. Amino acid comparison and phylogenetic analysis suggested that HGCRV might represent a new GCRV-I strain within the species group AqRV-C. Moreover, detection of HGCRV RNA copies suggested that the GCRV can proliferate in GCO, CIK, L8824, EPC, and GiCB cell lines. In grass carp, the RNA virus titer increased at first and then decreased, which is consistent with the absence of signs and mortality. These results provide a better understanding of the molecular epidemiology and genetic diversity of GCRV strains in China. Meanwhile, monitoring virus proliferation in various cells and in fish provided basic data for the accurate analysis of HGCRV.

## Figures and Tables

**Table 1:** Primers used for rapid-amplification of cDNA ends.

Segments		Primers	Sequence (5′–3′)
UPM		UPM-Long prime	CTAATACGACTCACTATAGGGCAAGCAGTGGTATCAACGCAGAGT
		UPM-short prime	CTAATACGACTCACTATAGGGC
S1	5′	GSP-S1-5	CCGGCAGCGAGATTCGCTAGTAGTCGG
		NGSP-S1-5	CGTTGAGTGAGCACACCAACGAAGGC
	3′	GSP-S1-3	CTACCCTAGGCCAACCACCCGTCCCTC
		NGSP-S1-3	CGGCAACGACGCCCAGATCAACTGCG
S2	5′	GSP-S2-5	GAAGACCCTGCGGAGCGGCTGGAGGG
		NGSP-S2-5	GACTTGCCTAGCCTGGGAGGAGGCG
	3′	GSP-S2-3	GGCATGTCCGATTCGGAAGCGCACC
		NGSP-S2-3	GTTGTGCACTTGTCCATACCCTCGTC
S3	5′	GSP-S3-5	GTCAAGGTATGCGGAGAGTGCCTTGG
		NGSP-S3-5	GCGACGGACGCGATACTATCAGAAG
	3′	GSP-S3-3	GCTGGCGATTGGGTATATCCAAGCGACG
		NGSP-S3-3	CCCGACGTGTCCACTACGCCTTCACCTC
S4	5′	GSP-S4-5	GTCGAGGGAGTCCAGGAGAGGACCAG
		NGSP-S4-5	CTCGAACACGAGTTGGTTGAACGCTGC
	3′	GSP-S4-3	CTCGCCGTACTCCAAAGCGTCATGGG
		NGSP-S4-3	GGCTGCTGCTGCTGAACGTGATCAAGC
S5	5′	GSP-S5-5	CAGCGTCGACATCGAGGAGGAATGTGCG
		NGSP-S5-5	TAGCCACTGGGCGAACCGTTTGGGC
	3′	GSP-S5-3	CGTCGTAAATCTGCTGGCACGTCGA
		NGSP-S5-3	TCGACGGTGACCCACCCTTGTCAG
S6	5′	GSP-S6-5	GCATGCGAGCAGCAATAGTGCGTTGC
		NGSP-S6-5	CGCGCATGTTCTCGTTGACGAATGAG
	3′	GSP-S6-3	TCCAGTGCTGCTTCGAGAGACCACG
		NGSP-S6-3	TTGGTGACGCCATCCCAGTAGCATC
S7	5′	GSP-S7-5	CGGGCTCAATGTGGCGCTCATACGC
		NGSP-S7-5	GCTGGGATCTCTACCACCTGGGCGG
	3′	GSP-S7-3	CGTTCAATATGACTCCACGTGGAGC
		NGSP-S7-3	CCACCGTCGACAACATCCGCTGCAT
S8	5′	GSP-S8-5	GGACTCCGATGTACGCCATGAGCGC
		NGSP-S8-5	GATGGCTGGTGATCTGACCACCGGAG
	3′	GSP-S8-3	CCTTAAATGGAACGATGGAGCCCGT
		NGSP-S8-3	GCGGCTAGACACCTGCAATGGCGTC
S9	5′	GSP-S9-5	CCTAACCTATCGGCATGAAGCAGGG
		NGSP-S9-5	GTCGACGGGCATTTGGGCGAGGTGAG
	3′	GSP-S9-3	CCCTGCTTCATGCCGATAGGTTAGG
		NGSP-S9-3	GTGGCTCGGCCTCATCTGCGGTCTC

**Table 2:** Characteristics of genome segments, predicted functions of proteins, and conserved terminal sequences in HGCRV.

Genome Segment	Gene					Protein				Predicted Function	Conserved Terminal Nucleotide Sequences
	Length (bp)	GC%	5′ NCR (bp)	3′ NCR (bp)	Position of ORF (nt)	Length (aa)	Coding Potential	MM (kDa)	Isoelectric Point (pI)		
S1	3949	56.32	12	37	13–3912	1299	VP1	141.26	5.85	Core protein, guanylyltransferase	5′-GUUAUU…UCAUC-3′
S2	3876	54.28	12	39	13–3837	1274	VP2	141.24	8.22	Core protein, polymerase	5′- GUUAUU…UCAUC-3′
S3	3703	55.98	12	46	13–3657	1214	VP3	132.15	5.88	NTPase, helicase	5′- GUUAUU…UCAUC-3′
S4	2311	60.28	26	65	27–2246	739	NS79	79.17	6.30	Nonstructural protein	5′-GUUAUU…UCAUC-3′
S5	2239	56.10	17	35	18–2204	728	VP5	80.35	7.69	Core protein	5′- GUUAUU…UCAUC-3′
S6	2039	55.66	30	62	31–1977	648	VP4	68.70	5.35	Outer capsid protein	5′- GUUAUU…UCAUC-3′
S7	1414	56.51	13	70	520–1344 14–484	274 156	NS32 NS17	31.70 16.90	5.72 8.21	Nonstructural protein	5′- GUUAUU…UCAUC-3′
S8	1298	58.32	12	47	13–1251	412	VP6	44.37	6.66	Core protein	5′- GUUAUU…UCAUC-3′
S9	1128	58.78	31	38	32–1090	352	NS38	37.95	7.21	Nonstructural protein	5′- GUUAUU…UCAUC-3′
S10	910	57.58	30	49	31–861	276	VP7	29.81	6.36	Outer capsid protein	5′- GUUAUU…UCAUC-3′
S11	821	58.95	43	43	44–778	244	NS27	26.53	6.75	Nonstructural protein	5′- GUUAUU…UCAUC-3′

**Table 3:** Comparison of nucleotide and amino acid sequence identity between HGCRV and other GCRV strains.

Segment	HGCRV		GCRV—I				GCRV—II							GCRV—III	
			GCRV-873		GCRV-GZ1208		GCRV-HZ08			GCRV-GD108		GCReV-109		HGDRV	
	bp	aa	bp (%)	aa (%)	bp (%)	aa (%)		bp (%)	aa (%)	bp (%)	aa (%)	bp (%)	aa (%)	bp (%)	aa (%)
S1	3949	1299	3949 (76.85%)	1299 (90.30%)	3949 (76.48%)	1299 (90.76%)		3927 (41.76%)	1294 (29.84%)	3928 (42.07%)	1294 (30.25%)	3929 (41.99%)	1294 (29.71%)	3943 (44.09%)	1294 (32.11%)
S2	3876	1274	3877 (77.96%)	1274 (94.35%)	3877 (77.60%)	1274 (94.35%)		3870 (50.97%)	1273 (45.85%)	3867 (51.32%)	1273 (46.67%)	3867 (50.86%)	1273 (46.43%)	3864 (48.32%)	1274 (43.39%)
S3	3703	1214	3702 (78.89%)	1214 (96.71%)	3702 (79.77%)	1214 (97.03%)		3753 (45.65%)	1232 (35.74%)	3752 (45.68%)	1232 (35.58%)	3753 (45.62%)	1232 (35.91%)	3729 (45.79%)	1224 (36.30%)
S4	2311	739	2320 (75.02%)	742 (79%)	2320 (75.17%)	742 (78.33%)		2263 (40.67%)	716 (22.65%)	2263 (39.98%)	716 (22.52%)	2263 (40.53%)	716 (23.19%)	2210 (39.29%)	715 (23.37%)
S5	2239	728	2239 (75.66%)	728 (85.03%)	2239 (75.88%)	728 (85.99%)		2229 (42.78%)	726 (26.61%)	2230 (41.75%)	726 (26.65%)	2143 (42.26%)	726 (26.77%)	2003 (42.18%)	638 (29.40%)
S6	2039	648	2039 (77.55%)	648 (90.43%)	2039 (77.64%)	648 (91.05%)		2030 (44.59%)	650 (32.63%)	2028 (44.64%)	650 (32.63%)	2029 (44.59%)	650 (32.48%)	1912 (36.46%)	609 (28.46%)
S7	1414	274/156	1414 (73.82%)	274/146 (72.53%/71.92%)	1414 (73.61%)	274/146 (71.79% 71.92%)		1604 -	512 -	1604 -	512 -	1605 -	512 -	1581 -	511 -
S8	1298	412	1296 (78.97%)	412 (90.29%)	1297 (79.28%)	412 (91.02%)		1560 -	361 -	1560 -	361 -	1560 -	361 -	1319 (39.39%)	418 (24.94%)
S9	1128	352	1130 (78.67%)	352 (91.48%)	1130 (79.56%)	352 (91.76%)		1320 (40.59%)	418 (22.86%)	1320 (41.24%)	418 (22.86%)	1320 (40.59%)	418 (23.12%)	1141 -	354 -
S10	910	276	909 (71.04%)	276 (69.57%)	909 (71.06%)	276 (70.29%)		1124 -	345 -	1124 -	345 -	1124 -	345 -	1122 -	346 -
S11	821	244	820 (74.91%)	244 (77.46%)	820 (74.04%)	244 (77.05%)		1027 -	310 -	1027 -	310 -	1027 -	310 -	876 -	75/140 -
Total genome lengt	23688	7616	23695 (71.04–78.97%)	7609 (69.57–96.71%)	23696 (71.06–79.77%)	7609 (70.29–97.03%)		24707 (40.59–50.97%)	7837 (22.65–45.85%)	24703 (39.98–51.32%)	7837 (22.52–46.67%)	24620 (40.53–50.86%)	7837 (23.12–46.43%)	23706 (36.46–48.32%)	7598 (23.37–43.39%)
Average			76.30%	84.09%	70.92%	84.28%		43.86%	30.88%	43.81%	31.02%	43.78%	31.08%	42.22%	31.14%

## Data Availability

The data that support the findings of this study are available from the corresponding author upon reasonable request.

## Supplementary Materials

The following are available online at https://www.mdpi.com/article/10.3390/v13040690/s1, S1: Genomic DNA sequences and deduced amino acid sequences of the healthy grass carp reovirus (HGCRV).
