# Supplementary material for: Isolation, Identification, and Genomic Analysis of a Novel Reovirus from Healthy Grass Carp and Its Dynamic Proliferation In Vitro and In Vivo
Source: Viruses. 2021 Apr 16;13(4):690. doi: 10.3390/v13040690 (PMC8073260; doi:10.3390/v13040690)
Supplement: Supplementary file 1 [file viruses-13-00690-s001.zip › viruses-1150225-supplementary.pdf]

Genomic DNA sequences and deduced amino acid sequences of the healthy grass carp reovirus (HGCRV)

Genomic DNA sequences

> S1

GTTATTTGCATTATGGCTGCGGTTTTCGGCATTCAGCTCGTACCTAAACTCAACACTTCTACGACT
CGTCGTACATTTTTACCACTCAGATTTGACCTTCTCCTTGATCGCTTGCAGTCCACCAACTTACAT
GGTGTATTATACCGAGCCCTAGATTTTAATCCAATCGACAGGTCCGCTACCGTCGTACAAACCTTT
CCCCCCCTTAATGCATGGTCCCCACACCCATCATTCGTCGAGAATCCGTTCGACTACCGCGATTG
GACCGACTTCATTCATGATCGCGCGCAAGCCTTCGTTGGTGTGCTCACTCAACGATACCCTCTAA
CACAACACGCTCAGCGCTATACTAATCCTCTTGTGCTTGGTGCTGCTTTCGGAGACTTTCTCAATG
CGCGTAGCATAGACATTTTCCTGGAACGTCTCTTCTATGATCCAACACTTGAGTCACCAATCACT
GCCATTACTAACTTCCCCTATCAATGGACTATAGATTTCAATGTCACCGATGACTCGATACGCACC
CCCGCAGGATGTAAATACGTAGCACTCTACGGCTACGACCCCTCACGCCCCGGCACCCCAGCAA
CGTATGGCAAACATCGGCCCACTTACGCTACCGTTTTCTATTACTCCACTCGCACCGCGCGCTCCC
GACTACTAGCGAATCTCGCTGCCGGTCCGACTGTCCTTGAACACTTCGACTCCCCCACATACGGC
CCCCATCTGCTAGTTCCACAAACTGGCGACGTTCTGGGTTACAGCTCGAGCCTGATCAGCCAAG
CCGCTCTACTCATGGTGGAGTCAGTCATGGATGCCCTCCGGGACAATGCTAATGCCAGTGCCTCT
ACAGCTGTCTCCCGCCTAGATAACGCTTACCACCCAGTCACCTCCTTCGACCCGACATCCTTCGA
CACGTTGTTGCAACGTGCCACTAACTTAGCTCTGCTAACCGTCCAAGGCGTCCAGTCCGAGAGT
GCCATCCCCCTGGTTCCCACGATGTCCGATGTCCGCAGTTTCGTGTCACGTCTTATGGCTGAAGG
CGATCCCCAGAGATGGTTCCCTTATCGTGTGGATCAGATACTGCATTGGCCCGAGTCGCCCTTCAT
TCCACCCATAGGTCCATTCTACGCGCCATTCAGACCCGTTAATTTCCCATTCACCACCGGCACCTA
CACCGTCGTCCCTGACGCAACCCGGCCTCTGCGCCTTCTACCGCAGTACCGCAACGCTACCATTA
CCATTCAACAAGCCGATGACGCTTATGATGACACCGCGCTTTCACCCCTAATCGTGAATCACGGC
TTCTGTGTTACCGGCGGCGTATTCACCTCTATTCACGACATCTCAGGCGATCCAACTGCCTTCCCA
GCGGGTCAATTGATTGATACTCCCAACACCTACTTCGATCGTGACCGCATGGCCCGACGTGACCT
ATTCCGCCGCCTCCGCGCCCCTGCCGATCGGTCAGCCATCAAGGATCGTGCCGTTTTCGACTTCC
TGGCTTCACTCGTGAACCCTACTACAGCCAACCCCGTCCTTGACACAGGTTTCTCCATGGCATAC
CTTGGTGCTTCCTCCGCTCATGCCACCGCCGATGAACCCGTGATCCTAGCCGACATCCGAGCGGG
TAACATTCCTGGTTTGCCCATCCCCCGTCGCATCGTCCAATTTGGCTATGATGTGGTCCATGGCTC
CCTTCTCGATCTATCCCGCGCCGTTCCGACGGGCACGTTTGGACTGGTCTATGCCGACCTGGATC
AAGTGGAGGACGCTGGCACTGATATGCCTGCCGCTAACCGCGCCGCCATCGCCATGCTTGGCAC
CGTCCTTCAGATGACTACTGCTGGTGGCGTTTCCGTCCTCAAAGTCAACTTCCCTACTCGCGCGTT
CTGGGTTCAAGCGTTCAATTCCTATGCGACCCATGCTACCACCCTCCACCTCGTGAAACCAACTA
TTGTCAACTCAAGTGAGGTGTTCCTCGTCTTCGGCGGCCGTCAGTCCAATGGTGCCCTGCGATCC
ACTACCGCTTTGCAGCGCTCCCTTCTCTCATTGTACGCCCGCAATGAGGCAATTGATCGCGCCGT
CGCTCACCTCCCTTTCTTCGGCGTCCCGGATGACGGTACCTCGGATCTCGGCATAGACGCCGTGC
GTCTTTTCGACCCCATGTTTTCTGACGCCGTTGCGAACCTCCCGTCCAACTCTCTGGCCGCGCTCG
TTAGTCGCGTCGTTCCCTCCTCGATCATGTTAACGCGCGTTCCATCCAACGGTCCGGTTTCTACCA
CTATATATGGGAAACGTACCTTCTTGAGCAATCGACGTCGTGCCCGCCTCCGTGACATCCCTATGC
TGATAACCACCACTCTGGTACACCAGCGACGATTCACTACCCCCCCGTCCTTTACCCTCTTCGGC

TCCGAAGCCGTCCCCGTCACCACTCTCGTCGCTGCCGGTTACAACTATTTCATTGCTGAACAGAC
GCGCAATCCCAATCTGGCGCACCTTCTGGATCTCGGTACCGGACCCGAATGTCGCATTCTCTCAC
TCATTCCCCCTACCTTGCAGATCACCATGTCGGACGCGCGCCCCTGCGCTGAGCTAATGGCCTCC
TTCGATCCCGCCCTCACCGATTATATTCAGGGTGATTACTCAATAGCCGCTTTTTGGAATGGGGTC
CGTTGCGATAGTGTCACCGCCATTTTCACCATTGGGGCTGCCGCCGCCGCAACGGGAATGGACC
TTATCGCCTTCCTCCAACAACTGATACCACGAATTGTCGCCGCTGGTGGCACTAGAATGTGGCTC
CAACTTAACACGCCCTTGTACGGCGCGACCTCCCTACCGGACCTGATTGACATTGATACACGAG
ATCACGTCTATCGCTTCAACGGCGGCGAACGAGTAGAACCATACACTGACCCCATTCCGCTGCA
GCAGGCGATTGCCGCCCTCCTACCCGCCGCCGCCCTGTCATGGCACTCCCTGTCCCCCACTTGCG
ACTGGCTATCATACATCATAGGTGTCGGTTCACCCATCAACCTCTCAGACATCGACACAGCGGTC
GCCTATTCTCGGTTAACGCCCATCCTCCACATAGACACCACCACCCCACCACTGCGCGTTAACCC
CGTCCCGACGCCCCTGAACCAGCAGTGCGCCATTCGCATCACATCACTTGACCCCGCCGCCGTC
CTCTCGGTGCAACACAATGGAGTCGAGGTCATCGGGGGTACTCCTGGGAACGTCGTCACCGTCG
CCGGTGCTGCAGTACTGCAATATGTGCCCGCCGCGCAAGAGTTCCTCCTCCAGTTTACCCCGACC
TTACCCGGCATTTTCGATGTGTTTCTAACTACCCTAGGCCAACCACCCGTCCCTCGTGGGTCCTTC
ACCATCTCCCCACCACCGACTACCGTGACAATTAACGCTCCAGCCCCCGGACAACTTGACTTCA
CTGACAACGGCAACGACGCCCAGATCAACTGCGATATGTATTACCAGCTAGCCGTGTGCGTCTTT
AAGGATGGCCAATACACCCGCGTCAACCCGGAGAAAGCCAGCGTCGTGACCAATGGAGCGAAT
CGTGACCTGCATTTTGTCCTCGACCTCTCCGACAACCACGTACTCCTCTATCTGTGCGACGTCACC
CCCTCCAGCCTCGGAGATCGCATTGCTTTCCCTCTCGTGGACATATACCGCGTCGCCTTCCCGAA
CAATACCCCAGTGCGTGCATCCCTCCCATATACCGGTGGCGGCGCCCATATCACCCAAGGAGGTA
ACCCATATCTATCACTAACGACCCCTCCAGCAGTACTACCCGCCGGCGTCGCCCTCGCTGCACTA
TCTACTTCAGTCGCTACACAATACCCCACCTACACCCTGCCCCCCGGCGCGTACGTATATGTGATT
GAGTAACGAGCGCCCACCTCCGTCAGCCTGCAAGCCAATCATC

> S2

GTTATTTGTACCATGGAGGAATTGTTCAACGCCCTACCTCAACCACTACAACAACTTTCACTAGC
CCTAGCCGGTGAGATACCACTAACAGACCACATTTTCGAACAAGCTTCATCCACCTGGCATGTCC
AGCCCCGCTCACTTACTTATAAATTACTAGATCATATCCAGTTTTCCACCCCCGTCGTCGTTCCGC
CTTCCATCTATAACTCGGTTGACTGGTCTAAGTGTTTCACCATCAACCAGGACCGCGTCGAGCGT
GTACCTACCATCGATGATCCTGACGACGTGTACGTCCCAAATTCAGACATTGGCCCCCTCCTTTC
GCCCTTACACACTATTCCCGACTATGGCTTCCTGCACCCTACGATTGAGAACGACGCCACCACGC
TCCGAGCCGAGCGCGCCCGTTGTGCCTCCACCTTCTACAAGATCGCCTCCTCCCAGGCTAGGCA
AGTCAAACTGGACCCCATCCGCATGCTCGGCTTCCTTTTACTCGTCCAATGTCGTCGTCGCGTGC
CATCAGGACTAGTCACTGACCAACCCTCCAGCCGCTCCGCAGGGTCTTCCCCTTCTCTCCACGCC
ATTTGGCAGATCATGCAGTATTATAAGGTTCAAGGGACCTACTACGCGCCCGCTCTTGTAGTGCC
ATCCGGTGCCATCTGGTGGATCCCACCCCCCGGCAAACGTAACATCGTCTCCGTTCAGTACCTGC
TCACCGACCTCATCAATCTTGCGATCCTTGCTCACATGACCGACATGTCCCCGCAGCTTGAACTT
GCTGGCGTCCTGATGTACCTGCGCGCTGCCTCGTCACATAGCTACGCCTACACGTTACTGCAAAT
GAAAGGAGTATTCCCTGCTCTCTCACTGCGCTCAATGTATAGGAACAAAGGTTTTGGTGGTAAAG
CCCCTGCTATCGAGTGGACCGAACCACGCTCCAAGTACAAGTTCAAATGGACAGGAGTCACTCA
TCTTCACGACGGCCTGCGCCCGCGCTCACCTGCCATGGACGACGCTACCATCGCCACCCTCTCG
AAATACGAACTCGTCGACATCGGCCACATGGTTATCAATATCCGCAATCAGCATCCTCAACACAA
CCACGACTCCGTGCGTTTCGTCCGAGACACGATGGCATTAACCAGTGGTATGTACCTGGTGCGTC
AACCGACCATGTCCGTCCTTCGCGAGTACTCCCAAGTTCCGGACATTAAGGAACCTATTCCTCCC

TCTGCGTGGACTGGCCCGATTGGCAACGTTCGATATCTCTTACCGTCCGTCCAAGGACCGGCTAG
GCATCTATACGACACATGGAGGGCAGCAGCCAGACAGATCGCCCAAGACCCCCAATGGCATGA
CCCATTAAACCAAGCTATCATGCGTGCCCAGTACGTCACCGCTCGAGGTGGTTCAAGCGCATCCC
TCAAATTCGCCCTGAAAGTCACTGGCATCGTCCTCCCCGAGTACGACGATTCTAAGGTCAAAAA
GTCGTCCAAAATCTACCAGGCAGCCCAAATAGCGCGAATTGCCTTCATGCTCCTTATTGCCGCTA
TCCATGCGGAAGTGACGATGGGTATCCGTAATCAAGTACAACGTCGAGCGCGATCCATCATGCCT
TTGAACGTCATACAGCAAGCTATCTCCGCCCCCCACACCCTCGTTGCTAACTATATTAACAAGCA
CATGAACCTGTCCACCACCTCAGGGAGTGTCGTTACCGATAAAGTCATCCCCCTCATTCTGTATG
CCTCCACCCCTCCCAGCACCGTCGTGAACGTTGACATCAAGGCCTGCGACGCCTCAATTACTTAC
AACTACTTCCTGTCCGTCATCTGTGGTGCCATGCACGAAGGCTTCGAGGTTGGTAATGCTGACGC
TGCATTCATGGGCGTACCCTCAACTATCGTATCGGACCGTCGCAGCCCTGTCGCCCCTTACTCCA
GACCCATTTCGGGATTGCAGACGATGGTGCAACATCTCGCTGATCTTTACGCGGCTGGCTTCCGC
TACTCCGTCTCTGACGCTTTCTCAAGTGGTAACAAATTCTCCTTTCCAACGTCCACCTTCCCCTCA
GGTTCAACCGCTACTTCGACGGAGCACACTGCTAACAATAGTACCATGATGGAGTACTTCCTGAC
CGTACATGCACCCTCCCATGTCACATCGCCTTCCCTTAAACGTATTTTATCGGATATGACCATCCA
GCGCAACTACGTGTGTCAGGGTGATGACGGTATCTTGCTCCTCCCGCATGAGGCTGCGTCTAAGG
TTTCTGCTGAGGACATGACTGAACTATTGGACTGCTTGTCCCAGTATGGCCAACTGTTCGGCTGG
AAATACGACATCGACTGGTCCGACACCGCTGAGTATCTAAAACTCTATGCCCTGATGGGGTGTCG
TATCCCGAACACGAGTCGTCACCCTCCCGTCGGTAAAGAGTACGCCGCACCCCAGACAGATGA
GATCTGGCCTAGTCTTATCGATATTGTGATCGGCCATCACTTGAATGGAGTGACCGACGTCCTTAA
TTGGAGAGAATGGCTCCGATTCAGCTGGGCATTCGCGTGTTACTCCTCTCGCGGAGGTTATACTA
ACCCAAGGGGTCAATCTTTCTCCGCCCAGTACCCATGGTGGACATTCGTTTACCTCGGCATTCCC
CCGATTCTACTTCCTGGCCAAACCCCTTTCATCCACTCGTGCTACATGCCCCCAGGCGATCAAGG
GATGTTCTCGATTCTCAATGGCTGGAGAGATTGGCTCATTGCACACGCGAGCAGTACTCTGCCTC
CCTTGCGACACCCTCATCCCGTGTGGGGTCTGGCTAATGTACCGTCCGCGCTTACTCAATTCGGC
GTCTACGCTGGGTACCATGCCGCACAACACTACAGACGCCCCAAGCCCGCCCCAGAGACAGCC
TCATCCGACTCTATTAGCCAGATCACGTCCGATCTAACTGAGTACTTATTCTATGACTCTGCACTC
AAGGCACGCGTCATGAAAGGTCGTTACAACTGGGACCGCATCTCCACCTCTCTATCATTGAATGT
CGGGTCGCGCGTGCCCTCCCTCTTTGACGTGCCTGGCAAGTGGGTCGCAGCAGGTCGTGATGCC
GAAAAGCCCCCACCCTCGTCCGTAGAGGAAATGTTCACCTCGTTGAATCGGTGCATCCGGCGCC
CTGCCCAGGCCTTCTCACGCCTCTTGGAGCTGTACCTAAGAGTACATGTTGCTCTCGGTGAATCG
ATCCCGTTGGCCATCGACCCTGACGTACCCCAGGTAGCCGGCGCTGACCCAGCTAATGATGACC
ATTGGTTTAAGTACACCTGTCTCGGTGACATCCCCTCAGCCACGAGGAACTACTTCGGAGAGTCG
TTGTTTGTCGGACGTGTCGTATCAGGTCTAGATGTCGAAGCCGTGGACGCCACCCTCCTCAGGTT
AAAGATCTTAGGCGCTCCTCCCGAGGCTTTTATCGCTGTACTTAACGGTATAGGCATGTCCGATTC
GGAAGCGCACCAGATAGCAGGACGGATCTCACTAGCAAACGCCCAGTTAGTTCAGATCGCCCG
CGTTGTGCACTTGTCCATACCCTCGTCATGGATGACGCTCAACACCGGACCATACATACATCATC
ATGCGTACGACTTTAAGCCCGGGCTGACTCAGCCGTCCGCTAAATCGCGTGACAAATCAGTGTG
GATGTCCCCGATCCTCAAGCTCCTCTGCACCAGTTACGCCATGACGGTCGCGGGCCCAGTGCGT
ACTTCCATCGTTACGGAAATTGATGGTTCCGCCGCGGCGCTTAGTGGTAACTTACGGGTGTGGAT
GCGTGATGTGTAGTGGTTGACCCTTGGCGTCCTGCATGGTGCTCAATTCATC
> S3
GTTATTTCCACCATGCCGCGCCGATCAGCCCGTAAAGCTCAATCTGCCACTAACACTTCCGCTGA
CACGAACGTTGTGCCTGCCACTGACGCACCGACCAGCAACTCACCACCCACTACCATCTCACCA

ACCCAAGCCGCCGCTGATGCTAATCAACAGCATGCCGGAATTTCAACTTCACAGAGTGGTCCAA
ACGCAGTCGGAGACTCAGCCCCCTCCACCTCAGTTAACAACGATGGAGATATCATCACCCGCCC
GACTTCTGATAGTATCGCGTCCGTCGCTAATGCTACCAAACCCGCCGCTGTCGTCTCTGACCAGC
AGGCCATGAAGGTGACCCCCATCGTCAACCCGTCCTCATATGTGTGCAACGTCTGCAACGCGCG
TTTTTCCACGATGTCCGCCCTCTCGGAACATTTGCGGTCCGACCATCGCGACGACGCCTCCACCC
TGTTAGCCACTCCTATGATTAACAACGCCATTCGCTCTTTCCTTACAGCATGGGATGGTTTGCGTA
TCCTAGCGCCAGATATATCCTCCAAGGCACTCTCCGCATACCTTGACAGCGCCGTCGCTAATGGT
CCAGAACTGATCATTGAGGACACTGGTCTCTGCACTTCTTTCATGTTGCTCGATAACATTCCGTCT
GCACACCTGACCGAGGAACTGATCGGATTCACCTGGTTCATGCAAATGTATCAGATGACACCCC
CCCTCCCCGAAGGTGCTGTCAACCGCATCGTATGCATGACGAACTGGGCATCCCTGGGTGACGA
AGGTCGCGGATTAGAAGTGCGATTACCCCCTCCAACCGACAGCAGCGTCCACGCCTACAAAAC
CGTCCTGTCCCGTGGATACATTGACAACGTCCAGTTCAATCCCCTCGCCCTGAGATCAAACGTAC
TCTTAATGCTGCTACAGTTCACTCTATCCAACCTGAAGATCAACAAGTCATCGACATTTACCTCGG
ACGTCACTACGCTCACCTCTGGCCGCATGATTCGCGCTTTCGAGGGACGTACCGAGCTTTTGGCC
CTAGCGTATCCAGGACGTGCTGTCCTACCGACGCAGACTAAGAACGCACAATTCCTCTCCACCG
CCATTCCTGACAGAATTGGTCGTCTGGATCGTGCCAATCTTATAGGTGGCGAAGTCAGCGCTATG
GTCGAGTGTATGGAATTGTGTGACGCTCTAACGCTAAACATCCGCGAGACGTACGTCATGCTGCT
TCGCTCCATGCACCAGGATCCCACGCAGATCGTCCAAATAGTCAATGAATGCGCGAACAACCTT
CTCAACTCTACCATTCCCATCAGCCTTCGCCCGACTATTCTCTGCCCCTGGTTCGCCTCATCTGAA
GATCTACGCCTCCAGCAAGTCATGCACCTTGTCAACATCTCCTCCAACACGGCCGCTGCACTCCC
ATTAGTGGAGGCCCTGTCTACCCTCCTGCGATCCGTGACGCCGTTAGTCCTCGACCCTACCGTCCT
AACCAACGCCATCACGACCATTTCCGAATCCACCACTCAGACAATCTCGCCGATTTCAGAGATC
CTGCGTTTGCTGCAACCCATGGGAAACGATTATGCCGCATTCTGGAAATGCATTGCCAGTTGGGC
GTACAACGGTCTCGTCACCACCGTCCTCTCCGAAGAGGCCTTCCCCGACAGTTCCCAGTCCATC
ACTCACCTACCATCCATGTGGAAATGCTTGTTTCTCACCCTCGCCGGACCCATGACCTCCGACCC
TCATTCTCCCGTGAAAGTGTTTATGGCCCTCGCCAACCTGCTCGCCCAACCAGAGCCCATCGCGA
TCGGCGTGCCCGGAATGCATCAGACGACCCCCGCTTCCCAGTTCTCTCACCCAGGAGTCTGGCC
ACCCGGTTTCCTCAACCCTCAACTCATCAACCCACAGCAGGCTCCCTTGCTGCGCGCCTTTGCCG
AACACATTAGGGCTAACTGGCCGCAGCCAAGCGAATTCGGTTACGGCAGCACGTTGCAGGGCTC
AGCCAACCTCTTCATTCCCCCTAATCGCATGGTGTACCCTTGGCCTAATCAGCCTCTCCCTCGGCT
AACTGTTGCTCCGACCTATGACTCAGCCATGTCGAATTGGATCTCCACCACCGTCGCATTCTTCAT
TCGTGTCGTCAACTCAGTGAACATGACTGCCACCGTCAATGACTTGACCCGCCGTACTATGACCG
GTGTCATGACTGCAATGAGGCAAGTAAAGACAATGACCCCATTCTACATCCAGCACATGTGCCC
GACTGAACTTTCGGTCTTAGCGTCCGTTACCGTCACGCCACCCTTTCAAGTGCCCTTCACCCGCTT
AGTGCAGAATGACGTCATCACCAACGTACTCGTAGCGCGCGTTGATCCTGCCCAACGTGGGGAT
GCTGCTGTAGATATTCGTGCCACTCACGCGACGTTCGCCGCTGCGTTACCCGTGGATCCCGCTTC
CATTGTCGTAGCAATGTTATGTGGCCAGACGGAAACTAACTTGATACCATCTCACCACTACGGTA
AAGCCTTCGCCCCGTTATTTGCGTCCAACGCCATGTTCACGCGGAACCAGCGTGCCGTGATCACT
CGTGAAGCTTTCGTCTGTGCTCGCAGTGCCGTCGCCCAGTGCCAAGATGCCGGTTTTCTCGTCCC
CAGGCCCCTGGACGCCCTCCGACAGTTCGACGTGACGTCAGCCGCAGCCGCAGAGATTATGCA
CGCAGTCAATGACGCGTTCAAGACCGCCTTTGACCTGGACGGCGCACTCCTCGATGGTCTGGCT
CTATACGGGGACCCACGTATCGCTGACCTATCCGCTGCATATCTGCAATATGGGGGAAACGTCGT
CCGCGAGCACGTACCACCTGGCACCTCACACATCCATCGCGCCCTACAGCAAGTCGAGTCAACG
TTCATGGCCGAGATGAACTTGTTCAACGTCGCCCGTGGGGACCTCTATCTCGTGCAGACTGCTAC

TAACGGTAATTGGTCTCCCATGGCCCCCGTCGCTGCCCCCCCATTCGTACGCGGAGGCCCCAACG
TGCGAGTGGTCGGCCGATTCGGTACCATCGTCCCACGCCCCAACGGTCTGGAACCCCAACTGAT
CGATGATGCCAATGTTCCCCAAGATATTGCTGGCGATTGGGTATATCCAAGCGACGTGCTACAAG
TATCGGTAGCTGTGTTCCGAGATTATGTATGGCCTATGGTTAAGGCTGGCCGGACACGCGTCCTAA
TCGAACTGGGCCACTACGTCTACACCCTCCATTATTACGACCCTCAGATCAGTCTCGACGAAGCG
CCGATCCTGGAAGAATGGCTAACCCACATAAACCCGGCCGGCATACCACCTGTCCCCTTCTGCAT
CCCAACACCACAGGTCTACCCATGTATCACCGCCCGACGTGTCCACTACGCCTTCACCTCAGAG
AATAATAACGATTCACTATTCTCTACCAACGCCGCTTCGATCGACACAGCCTTCGGAGAAAACGC
CGCCGTCTCCCCCCTCCGCTGGCCCGGCCTCGTTGACCCCAACTACCGCGTGGGCACTAACGAC
CTCCCCAACCGCATCACCCTCTATAACTCCCTGTACAGATACAACTTCACCTACCCGACCCTCGA
TGGCATCATGTACGTGCGCAGCGCCACTTAATGGCGAGGTTGAGTAGGGCTCCCTGCGGCTCTTG
GTGCTTTTCATC
> S4
GTTATTGAAGAGCTTTGGAAGTAAACATGGCACGCCGCATTACTTTGAACAGTCTGAAACCTCTG
AGTGCGATGAACAGTCATCCCTCTCTGACTCCCACCACTACCCCCACCCCCGCGACCGCCTCAA
CCTCCGCCGCTCCTTCTAATGACTCCCCAGCCACCGCCTCCCTCCCAGCAGCGTTCAACCAACTC
GTGTTCGAGTTCAATGGCGTGAAGTACACCGGTTCCCCTCACTCCTGGATGATGCCATATCGTGG
ATTCGAGAAGCACATGGTCAATATGGTGCTCGACACCCGCCCCGACGCCACCTTTTCCCGCTTCG
ATGATCTCCTTTCCGCCATCGTATCCGCGCTTGCAGTTAATGGCGTCACGGCCACCACCCCCTTCA
ACGAAGGTGAGCTGATCCTGCTACGTTTTGCCTGCCTGAAGACACTGCGTGCATCCGCGGCCGC
ACCCACCCCATCCATCTCTGATTGGTCCGACTCCACGCCTGGTCCTCTCCTGGACTCCCTCGACTA
TGGCCTCCCACTTTCCCCCTCCTGTGAAGCCGCCCCTCCCGTCCTCGAACCCCTCCGCCACTCAC
CGCTACCACCCCTCCACCCTGATCCCGCCGTCTCCCACTCCCTCGTCCCCATGGATGTACCCGAC
TCGGTCCTTGTTCCCCCATCTCCTACGCCTGCTACCGGCGCCGTCATCCCCAGTCTGCTGAGCCTG
ACCCTCTCCACTCCGCCCCGCTCCCGCGCCAGCACCGCATCCTCCAGCTCCACCCCAGCCGTAC
CCCGCCTTTCCGCTGCTGCCACTCCTCGACGCCCAACTCACATTGCCGACGATGAGACATACAA
CCGGTCTCGAGCCGCCTACGCTATGGGAGTGCCCGCCCTGTCCCCAATGTACTGCACTGGTAAG
GAACGCCACTTCGAGCAAACCTTCTACTCCGCCTATCCCCACGCCGCAAACGGGGTCTGGACCG
CCTATCAACACTCCATCATCCTCATCGCCGCTCCCACCGAAGACATATCTCTACTAACACTGCATC
ACGTCGAAAGAGAACAGTCTGCAACTGAAGCTTACCATCTCCGTGCCCTCGATGGAACCAACGT
CGCTCGCGTTGCCGTCTGCCGCCTGGACCCATCCATGACTTGTCGTGACGTTCACGCCATGATGG
CTGGTCACAATGTCGTTTCCATCTCTGGTTTAGCCGCCGCGTTCATGATCCGCCACCGCCTCACCG
ACGGCGTGTTCAGCAAAGCCTTCCGCCGTATCGTCATGGGCATCGACCCCGTCATGATGAGACA
CGACCCCGTCCCCCTCCACCTCTTCGCCATTCTCACCGACCACCGTCTGGACCACGCTGCCACCC
ATGCTGTCATGTCCATGCGTATAGCGCTCATGCAGGTCGAGTCAGCTTCCTACCAGGCGACGAAG
ACTTGGCTCCGTGGACACCTCCCCGTCACTATCTTCGCATCAGCCACCACCGACTCATCCTCCGA
CTCCATTCACGCCACCATCCTGGAAGCTGACAAGGGAATGCGTACCGCCGATACCCCTGGCTCC
TCAAATCTACGCGAACTCGAGGCATCTAATGCCGCCCTCCAACGCCAGGTCATCGATATGGACGT
TCAAGTCAACGCACTCCTCCGCACCATCTCCGACCTCAAGAGCTACACCAACCATCAACAGGCT
TCTCAGGGACAGACCATCCAGCAATACCTACATTCCCACGTCTGCGTCAACACCAAGGAACTCG
CCGTACTCCAAAGCGTCATGGGGGAGCACGCAGCCAACGCGATCCAAGCAGTCCGCGTCCACG
CCAATGACACCGCCAAAGCCGCGCTCTCAGAAAAGGTCTCCGTGCCCCTCACCGCCCAACTGTC
CGACACACTCGCCCACCTACATGCTGCGCGGTCCCGCAACGATGAACTAGTCGCCCAGTTGACC
ATCGCCGAGTCCGAAGCAATGGCTGCTGCTGCTGAACGTGATCAAGCATGCGAGCTGGCATCGG

AGCTTCAGACTCAGGTCGCCGCCATGCAGCAGGAGTATGAAAACGTGACCCGCGCCCTTATGCA
GGATAACGAGCAACTGCAACACTCCGCCGCCACCGCCGAAGCAGCCGCCACCTCCACGTTCCG
ACCCACCCCCCCCCTCGTTTATGGAACCGCGCCCTCCGCTCTCCCCGCCGTCGGCCCAGGTCTCA
CCATCCCCCTCCTAGCTCCAGAAATCGACCCCGCTTCTCTATTCCTGTAATCGCCTGTGGTTTCTC
TGTTCGTGTCGTCGTGTCCCTCTGTTTCTCCCGCTCTCTCCCCTTCATC
> S5
GTTATTTGTAGCTAAGGATGATCACCATCGTGGTTATTCCAACAGCCCACTTTTCCTGGACCGATA
CCAACTTCTTAAATTCCGTCGATTATCGACTAACCACACAACCCAAAACTAGAGACCGTTTCGCT
GTTTACGCCCCCGGATGGCTTCGACGCCAATTGGATGAATTTTCAGCGTCCCTCACCGCTTCTGA
ACTCTTACAGGCCCTAGAATCTATCCCCATCCCCGTGAAAGCCCGCTGCCTGCTGCTACCTAAGC
CCAAACGGTTCGCCCAGTGGCTATTGGACGTTCCCGCTGCCAATCTCTGGCACATTCCCGTCACC
ACACTTCGCGCTACGGTCGCTTCCAAACATCCTTCCTCGGACATATTCAATTTCATCGCGGACCA
CGTACCTCCCAACGCCGAATTCGATACGATCACCAGACGCGTACCTGCGGGTAAGGATATCTAC
GTCAGGACCACTAAAGTTATCGGGGCTCCCCTGTGCCTAGCAGCACCAGCTAAGTACTACGCCG
GATATCTGTCAAGTCACCAGTTAGATTCCATTTACCCTGACGGCTGGTCCCCTGAGACGTTCCGTA
AACGAGAATACTGTTTAACCATTCTCCCTTCGCTGTTGGGTCCACGCACATTCCTCCTCGATGTCG
ACGCTGAACGTGATACCACCTATCCGCTATCTGTGCTCTGGCCACAACTTCGTGCCGCAGCCCTC
AAATCGCGACTATTACTACCTCCAGTGGCGTTGCTACGTCGTGTCGTTGACCCATCCCTCAAACCT
ATCTGGTCTGCTGATACAGATCCGGCTTTTCGAGCTTTACGACTATCTCGCCCATCCACAGCCTCC
AGGCCGGCCGGCTTTGATTTTTCTGCCCTTCCCATCGCCGACCTCATCTGCCTCTTCGAATCCGAA
CCCGACTCACACGGGCGCACCGCCCCCGGCACCAGGCTCACCCTCCATGCCGTACCTACTGATC
TGCTGACCGCTCTCTCGATTCAAGAGAATGTGCGATACCCATTGCGACAACAGAGTGGCATGTTC
GTACATTGGGTACTGTTGGCCCTACTTATGTCCGACGACGTTTCCGTCACAGGCACCCGCCGTTC
CATTAAGATGGAGACTGCTCACGCTTCGGCCAAGCCGTTTGTCCACATTACTATCGAACGGTGCG
TGTCAGCACGACTCATAGACGTGAGAGGATCAACCGCCATGTACGCAAACGCCGTATGTTTGAC
CTTACCCAAGGGCTCGTACAAATCAACGATCATTGACACCCTCCCGGCCATGTTCCCCGACCTAC
CGATTCTTGACTCCAATGCCGTCATCGACTCTGACGCCCTCGGCGACTCACTACGGCCGTCATTT
GAATTACAGTTCCTCGAGCGGCTCAATGTCCTGGACGCCGGTGTAATCGATCGTGCCGTCGCATC
GATTCTCAACCCAACCAGTGATACATCCGATGACGCAGCAGCGATGGTGCTCGAGACGTTCAAC
AGTCTCTACCGTGATGTCATGACGCCCGCTCAGCGCACCCGCCTTCCTTTCCTTACCCAACAGGG
ACGTGTGCTCGCCTTCGCCCACTCCGATTATGAACTCCTCGCTGCTAACATACCCATTCAAGTCAT
ACGCGGGGCCATACCAATTGATCACGTCGTAAATCTGCTGGCACGTCGAAATCGCGTCGGCGGC
ACTGCTCTCCAGGTACTCCTAGACTACTGCTACAGGACCCAAGCTTCTCCGCTTGCCACCCAGCC
AGCAGGTCGCCTGTACAAGCAGCTTTTCGGTCCCTGGCTAATGGTTCCGCGACTCGACACCCCG
CTCATCACACTACGCCTGACGGCGACCGCCCCTGCAAAGGTCTTACGCGCAGCTGGATGGACCG
TCGACGGTGACCCACCCTTGTCAGTGTCATGCCTGCGCGCCTACGTCACCGACCGCGAATCGGC
CGCTTCCCTTATTGAAGCACGTTTGGACTCACGCGCCCTAGTCACCATCGACGGCGACCAGATCA
TGTTCGTTGAGTACGCGCCACCCCTGCCACTCGTGTCAATACCACGCACGTTCCTACTGCCCGTT
ACATACGTGGCCCACTGGGTCGAACCCGAGCGCGTCCTATTACACGGTGGTAACGTGTCCTTCA
CCAGTGGCCTGGAGTGGACCTTTGCTGATGATCCTCAGGGTGTCACCCCGGCCGGGGTTTGAGC
AGGCCGGGGATCCCTGACGCTACGCCAATCATC
> S6
GTTATTTCGACACTCCGCACTCTCTCTACAATGGGGAACGTTCAAACCTCCGTCAACACTTACAA
CATTAATGGGGATGGCAATTCCTTTGCCCCCACCTCCGACATGACATCAACCGCTGCACCCGCCA

TTGACCTGAAACCCGGCGTCTTAAATCCCACCGGCAAGCTCTGGCGTCCAGTAGGTACTACCGTT
GCAACGGTTGATTCATTAGCCATCGTTAGTGATCGCTTCGGTCAATACTCATTCGTCAACGAGAA
CATGCGCGATACATTCGCGAAAGCCCTATTTGACATCAATATGTGGCAACCCCTATTCCAAGCTA
CCAAGACTGGATGCGGCCCAATCGTCCTCTCGGCATTTACCTCTACCACATCCGGTTACGTTGGC
GCTACCGCTGGCGACGCACTCGATAATTTCGTTACAAACGGCACATTCATCAGCTCGGTCCAAAT
CCTAAATCTGCAACGCACTATTGCTGCTCGCATGCGCGATGTCGCCCACTGGCAGAAACACTTG
GATACCGCGATGACCATGTTGACACCCGACATCTCGGCTGGTACCGCTTCCTGCAACTGGAAAA
GTCTGCTTGCCTTCGCTAAGGACATCTTGCCCCTCGACAACTTATGTCTGACGTACCCTAATGAGT
TCTACAACGTGGCCGTAAGTCGATACCCCGCCCTGCGCCCAGGTAACGTTGAGACCAAACTCCC
CGATGCCCAGTCCCATCCACTCGGCGAAGTCGCTGGAGCGTTTAATGCCGCCACGTCTGAAGTC
GGAAGTCTAGTTGGCTCTAGCTCCACTCTGTCGCAAGCGATATCCACCATGGCTGGCAAGGACCT
AGATTTAATCGAAGCGGACACCCCACTCCCGGTCAGTGTGTTCACACCATCTCTCGCCCCGCGCT
CCTACCGCCCAGCTTTCATCAAGCCTGAGGACGCCAAGTGGATAGCCGAATTCAACAACCCTGC
CGTTATTCGCCAGACCTTATCCTACGCCGGCGATGATTACACCATCCAACTTGGACCTGGACCAA
CTCGTGTCATCGACATGAATTCAATGATCGACGCCGTGCTAACCCTCGACGTGAGTGGACCCCT
CTCCCTTTCCAAACAAATGCGGATCTCTCATCAGCGATCCCCGCCTTTGTCCTCATCCAGACGTC
CGTCCCAATCCAGCAGGTTACCTCTGCATCAAACATCACCGCCGTCACTGTCATCTCCGCCGCGG
AAGCCTCCGCTATTAACCTGTCTGTGAATGTGCGAGGCGACCCCCGCTTCAATATGCTCCATCTG
CAAGCGACGTTCGAAAGGGAGACCATCGCTGGTATTCCATACATCTATGGACTCGGCACGTTCCT
CATCGCTTCGATCACCACCTCGTCAGATTTCTGCAATCCCACACTGATGGACGGTCTACTTACTGT
CACTCCAGTGCTGCTTCGAGAGACCACGTACAAGGGCGAAGTCGTGGATGCCATCGTACCGGCC
ACTGTCATGGCAAATCAGACATCGGAGGAGGTCGCGTCAGCGCTAGCAAACGATGCCATCGTGC
TCGTCTCCAATCACCTCACCAAGCTGGCCAACGTGGTTGGTGACGCCATCCCAGTAGCATCTAA
AACAGACGACTCCGCCACCAGCGCTATCGTGAGCCGCCTGGCAGTGCAACATAAGCTATCTCAA
GTGGGGCGCACCAATCCGACCCCCCCGGATTATCCTCTGCTATGGCGACGAGCTAAGCGGGCTG
CATCTATGTTCGTTTCTAACCCGGCGCTTGCCCTCCAAGTCGGAATACCCGTTCTGACACAGTCC
GGCATGCTATCCGCCCTCACGTCAGGCGTAGGCACGGCACTCCGCACCGGCAGTTTGGGAAAAG
GGGTAACGGACGCCTCCGAGAAACTTCGCGCTCGCCAAAGTTTGACGGTGGCCAAGCAGACGT
TCTTCGACCAGATAGGGAGCCTGTGGCCCGGCAAGTGAGGGGTGGGTTGACAACCCCCCTCCGC
GCCCGCTTGAACCGCGGGGGGTGTCGTACTTTCATC

> S7

GTTATTTGAACCCATGCCTTGCCAGGATACTGTTTCTCTATCTATACAGCACACATCGGTCTACGT
CCAGCATTCTTGTTGCGTGTCCACAACCACCTCAGCCTCAACTTCTGCTACCGCTCTTGGCCTCG
GTTGCATTGCCTGCGGGATCATCGGCGTTATCGTCGTAGCCAGTGGCCTGTGTTGTCTTGTCAATG
GTCGGTGCTCCTATTGCCGAGGGTTGGCTCTGCGGTCCAGTTCCTGGAAATCCCCTACAACATCA
CTGCGCACTCACCAACCTATCTCTTTCGGTCTTCGTCACCTTATTGGGACTGATCTTCGGCGCCCT
ACTCATTCTCGTTCTTTGGAGCTGTTGTCAGACATTCATCCTGTGGTTCCGAATCGAGAGTGCCCT
CCGCCATACGATTCGCTTACTGTTGAACCAGAAGTCCACACGCCTCAGACACTTCAACCCCAGC
CCAGACTACAACCGCCCAGGTGGTAGAGATCCCAGCCCCAACACCAATCCCTTCGTCTAACATG
AACACCAATCGCTCCCTTATCGACGAGCGCTACGACTGGATCGTTGCGTATGAGCGCCACATTGA
GCCCGAAGCTATCACCGCCATCCGTACTCCCGCGTACGGCGCCCTACCCCCAACCCAGATTCCA
AAGTTCCTAGGCCCCAACCCCAAATACTTTGTGTACGACGGAAACGTCGAGGAGGTTGAAGGA
CAACTGATCAAAGACCCTTCAGACGACTATCAACCGCGCCGCTACGCTGACACGTGGACATGCT
CTCTGTTCACGGAGGTCCCCCCCTCGCACCCAAGCTGGCCTCGCGACACTGTCGTTCAATATGAC

TCCACGTGGAGCCCCTATCACCATGTGCCGATGCGCTACTTCCCACGGTTCATGGGACTCGAAGA
TGACCTCATCAAACCGAACGACGGCTTCTGGCCAATCCTACACCGCTGCGCCGGATTTAGATTTC
GCTACCCGTGGCGATTCGACCTCGACGCACTCGCCGCCGGAATCGGAACCACCGTCGACAACAT
CCGCTGCATCGCCGAATCCGGTGCCTTCGCCACCGTCGTGGGTACTCTTCGCGCCATGGGCTGGA
AGTATCACCACCTCTCTGGCCTACGGCCCCAGCACGTCGAATATTGCGTATCCGCATGGGCATTAT
CTTTCACCCGTGAACAGCTCGACGATTATAAGCCCGGAGCCTGGTTCGATGACCTTCTGCGCGTC
CCAATCTACCCTGACGAACAGCCCATTGGCCTAAACCGTGAACAACTGTTCACTCACCCCTTCGT
GTTGCTGAGCTGGATCAGGATTCAGTTACTAGGTGGAGGCGGTTAAGCTGCGGGAAGGGTCCTT
TTTGACCCCGGGGTTCCGGCCACCGCGGCGGCGCCGGTTCGAAGCGTTCATC

> S8

GTTATTTTTGTGATGGCACAGCGTCAGTTCTTCGGACTCACCTACAACTTTTACGGCCAACCCGCC
CCCCTTTATGATTTGAACGATTTACAAGAAATTGCCGGTTGCTACGCTCGCCCCTGGACTAGCCG
GTTCAGTCACCTTGCCATTTCCACCGGGTCACTTCCAGTGTGGTCTGCCCGCTACCCCAGCGTCG
CCGCCAGAAACATCATCATCAACACTATCCTCGGCGCACATCTCAATCCTTTCTCCGGTGGTCAG
ATCACCAGCCATCAAGGTATCACTTGGCGTGACCCTGTGCTCTCTTCACTAGTCCCAGTCCCAGC
CGTTCAACCTCCACCAGCATGGGCGGTCGCCGAAAACGTAGCGTTGGATTCGAACAACTATCCT
ACATACGTGCTAAATCACTCCGCCATGTGGCCAGTCAACCAGGATGTACATGTGATGACCATGTG
GGCTTTGTCCGATCAAGGACCGATCTACCGACTAGAAGTCCCGGCCGCACCCATGCCTGCCGCC
ACGACTGCAGCGCTCATGGCGTACATCGGAGTCCCGCTAGCCCATCTGGCCCAAACCGCCTACA
AGTTCGCCGGTCAGCTGCCCCAGTCACCCGACTCAACTATGGTCTCTACCATCCGCTGGCTGTCC
GCAATATGGTTCGGGTCCCTCACTGGACGCCTCAACCGCTCTCGCACCTGCAATGGTTTTTACTTC
GAATTTGCGAAGCCCTCCTTAAACCCTGACCAGGCCATCCTTAAATGGAACGATGGAGCCCGTG
TCGCCGCCCCAGCCGCCGCCCAGGTGTCCTACATGAGATGCATTTCCCCCCACTGGCAGCATCA
GATAACGGAGGTCGCCGGCGCCATCATGAGCCAGTCCGTCACCGCAGTTACAGGTCTGGCCACC
CTGATCGATGAGGCCACACTTCCCGCGTGGTCCCAAGGCGTAGCTAACCTTACAGGTAACGGCG
CTGGCGTCACCCCTTGTCTGGATTACAATCCCGTTCCCATGGCGGCGGCTAGACACCTGCAATGG
CGTCAAGACGGACTAATAACTGCCGCCCAGGAAGCCCAGATGAATGCCGACTACACAGCCTAC
GCCCTTACCATCGAGCAACACCTCACAGCTATGCTCGCCGCCAATCCCGTAGCCGGTGGCCGCA
TGCCCATTCAACCGTTTGTTGCAGCTGACTTCGGCCAGGCCGGCCAAACTGCGGCGGCGGTCGC
TTTAGCGCAGGCGATGTTCGTCTAACTTGGGGTGGGCTGCTCCCCCTCCCCCGCTGCCTCACGCG
TTTCATC

> S9

GTTATTTTGAGCTTACTGATTGACAACCATCATGGCACACACAGGCACCGCTACTCTGATCAATG
CCGAACGCACTGACAGAACCCTCCGTCTTCTTGAAACTTTCCCTTCCCTGACAATCACCGTCCGC
CAAGACACGTCTGGACGCGACCTCATCTCCTCTACCTACTCCGCTACTGGCATGTCCGCTGCTGC
TCGCAACCTGAATCCAATCGCAAATGTTAAGAACATGCGTCAGCCCGGTTATGTCAAGCCCGCT
CACACCCCCACTAACACCGTCCCATTGCGAGTTGCTACCTCCACCGGCCTCGAGCTGCCCACCG
CTCACCTCGCCCAAATGCCCGTCGACCAGGCGCTCCGTGATGCCGTCGCCGCCGCTGTCCCTAC
CCACGCCGCACATGTGTTGCCCCCACAGGTGGACCGTGTCACACCGCTCACACTAGCCGCACGC
GTCGCGATGGTATGTGCTGGTCTCGACTGTGACGACATTCATGAGATACCCCCCGTTCCAACCGC
TATGGCCCTAGCCTTCACCACTAAAGTGCTACTGATCCATGTGGTCGTTGATGGTGACCGCGCGT
CCATCGCCGTAAACCCCGACCCCGCCCGTGCCCTGCTTCATGCCGATAGGTTAGGCAACATCATC
ACCAATTATGGATACGATGTGCGTGGAACCGTTCGCCGTGACGCCGCCGCAGCGCTCGCCCCAT
CAGAGTTACCTGCTGCCTACCCGATTGAGTGGCTCGGCCTCATCTGCGGTCTCATTGCTACCCAA

ATCGAGCTGGATCTCGACATGCTTGCCATGAACCAGACTGAGCAGAAACTCGTTGCTCCACACG
TGCAGGCCGTCGACCCATTTATCAACCGCCTGCAGAGCTACGCCACGCTTTCAGCCCGCATCATG
CACCTGTGCGTCACTCATTCCCAACGCCCGTTCCGCGACTTCCCTGAGCTGCTGAGGCACTGGC
AGAAACCCGAGCTTACCACCGCCATCCCTATTAACATCACTCTCAGGGGAGCCGCTCTCGAGGT
GTCCGGTAACGGTGCCGAATTGTTCCGCGTGAGGGCACTGCCAATCGGGGGCATGTAGAGCGTG
GTCACCGCTCCCATGCCGCTCATACTATCATC

> S10

GTTATTTCTGAGCTCCCAATCATCACCACGATGCCACTCCACATGATACCGCAGGTCGCCCACGC
TATGGTGCGTGCAGCCACCACCGGACGTTTAACTATGTATACCCAAACCCGCACTGAAACCACT
AACTATGACCACGCTGATTACATCACATGCGGCCGTTACACCGTCTGCGCCTACTGCCTCACAAC
CATCGCCCCACATGCCAATGTCCAAACGTTGCCATCAGATCACTTCTGCCCCCGTAAACCTAATG
AGATAGCCGCCACCCTAATCGAGGTGAGTGACAAAACGAGAGCCGCACTGGCACGCAGCCGA
ACCATTGATTATCACACCTTAGCCGTGCAAGCTGGATTTGTCGAACCCACCGACGACGACGATTC
GTCTATATCCGGCGATATCACTGAGCTCCCCTTCCGTACATGCGATCTGGACGTCGCGTCCGCCTC
AGCTTGCGTCCGCTCCCACTGCCAGGCCGGTCACGACGGGATCCACAGTCTGCCCATTATGACC
GGGGACTTCAAACTACCTAACGAACGCGTGCATCGCGCATGGGGAACCGGCCAATCCCAGAAT
AAGGTGTTGACGAGGTGCCCCACCACGGGTCTGCTTCTAGCCCACGACACCCAACTCCGTGCCA
CTTCCGTCGTCGCAACGGCCGCTACCCGTGCCGTGTTAATGCACAGCTTGCTGACGTCCGCTAAC
GCCTCTGACGGGCCGCAGACACGCGCCGCTTGCTACGGACCCTCTTTCAGCAACCTACCGTACG
CATGTCACTCAGCCTGTTCCGCCGATATGGCCCATTTTGATTGCGGCTATATCGTTGGTCTCGACC
TGCACGTGGAGCCATCCGATTAAGCGCCGGACTACCCACCCGGTGTAGGTGTAGGGGTCTCAGC
GTATCATC

> S11

GTTATTGTTCTCCTCCCTTGCGTACACGCCTCAAACTTCAAACATGGCCACCGCTGGAGTCACTA
ATGCGATTGACGGTCTCATCCTCAGTCGTTTAGACGAAGCAATTACCAATAACCCCACTATCCTA
CGCCTGCGCAAGAAAGTGTCCTCCCTGTGGAAGGCCTACGATAACCATGTCGCCGACCCAACGG
CACACGAGCTCCTCACTATGCCCGCGGACCTCGCAGAGTTACCCCCACCCCTCCCCCCCACTGC
CGAAGAACTGAACTGCAGCTATTACGCCACTGCCCCCCCCACCCCACCTCCGGCCGTAGATACG
CTCTGGGGCACCGCCAGCTATGAAATAATCGGTCGCGAGACGCGCCTGGCCTCCAGTGAGAAA
GCGCCCCGGATTATCATCCACGTGACGGACGAAACCATCCGCCTAGAGGTGCCCACAATGGCAT
TCACCCTCCCGAGCGCGTTAGCCACGGGTTCGCTGCTACGCGTGACGCTTCCAAAGGGAGCAAT
CCACTCAGCACTGACTGGAAAGTTCGATGAACGATGCTGGATTCGTCTGACCGCCACCCTCACC
AAATGGATGTCCCCCAGCACGGTCGTCACCAGCGACTGGACGCTCGAAACTCCCACTGGCCTCA
TCAAGCCACCAGTCACTGGCTTCAATGCACGTGCGACGCTCCGGGACTCATCGCTCGTCCTCTG
GTTCCCAGCCCCAAATACCTCTATGATAGCACGCCGCGCAATAGTGAGCCCCATGTCGTGCGTGT
TTGAATGAGCGGCGCAGGGGTGACCACCCAGGCGGGGAGGTGTCTTTCATC

Deduced amino acid sequences

>S1

MAAVFGIQLVPKLNTSTTRRTFLPLRFDLLLDRLQSTNLHGVLYRALDFNPIDRSATVVQTFPPLNAWS
PHPSFVENPFDYRDWTDFIHDRAQAFVGVLTQRYPLTQHAQRYTNPLVLGAAFGDFLNARSIDIFLER
LFYDPTLESPITAITNFPYQWTIDFNVTDDSIRTPAGCKYVALYGYDPSRPGTPATYGKHRPTYATVFYYS
TRTARSRLLANLAAGPTVLEHFDSPTYGPHLLVPQTGDVLGYSSSLISQAALLMVESVMDALRDNAN

ASASTAVSRLDNAYHPVTSFDPTSFDTLLQRATNLALLTVQGVQSESAIPLVPTMSDVRSFVSRLMAEG
DPQRWFPYRVDQILHWPESPFIPPIGPFYAPFRPVNFPFTTGTYTVVPDATRPLRLLPQYRNATITIQQA
DDAYDDTALSPLIVNHGFCVTGGVFTSIHDISGDPTAFPAGQLIDTPNTYFDRDRMARRDLFRRLRAPA
DRSAIKDRAVFDFLASLVNPTTANPVLDTGFSMAYLGASSAHATADEPVILADIRAGNIPGLPIPRRIVQ
FGYDVVHGSLLDLSRAVPTGTFGLVYADLDQVEDAGTDMPAANRAAIAMLGTVLQMTTAGGVSVLK
VNFPTRAFWVQAFNSYATHATTLHLVKPTIVNSSEVFLVFGGRQSNGALRSTTALQRSLLSLYARNEAI
DRAVAHLPFFGVPDDGTSDLGIDAVRLFDPMFSDAVANLPSNSLAALVSRVVPSSIMLTRVPSNGPVST
TIYGKRTFLSNRRRARLRDIPMLITTTLVHQRRFTTPPSFTLFGSEAVPVTTLVAAGYNYFIAEQTRNPNL
AHLLDLGTGPECRILSLIPPTLQITMSDARPCAELMASFDPALTDYIQGDYSIAAFWNGVRCDSVTAIFT
IGAAAAATGMDLIAFLQQLIPRIVAAGGTRMWLQLNTPLYGATSLPDLIDIDTRDHVYRFNGGERVEP
YTDPIPLQQAIAALLPAAALSWHSLSPTCDWLSYIIGVGSPINLSDIDTAVAYSRLTPILHIDTTTPPLRVN
PVPTPLNQQCAIRITSLDPAAVLSVQHNGVEVIGGTPGNVVTVAGAAVLQYVPAAQEFLLQFTPTLPGI
FDVFLTTLGQPPVPRGSFTISPPPTTVTINAPAPGQLDFTDNGNDAQINCDMYYQLAVCVFKDGQYTR
VNPEKASVVTNGANRDLHFVLDLSDNHVLLYLCDVTPSSLGDRIAFPLVDIYRVAFPNNTPVRASLPY
TGGGAHITQGGNPYLSLTTPPAVLPAGVALAALSTSVATQYPTYTLPPGAYVVYVIE
>S2
MEELFNALPQPLQQLSLALAGEIPLTDHIFEQASSTWHVQPRSLTYKLLDHIQFSTPVVVPPSIYNSVD
WSKCFTINQDRVERVPTIDDPDDVYVPNSDIGPLLSPLHTIPDYGFLHPTIENDATTLRAERARCASTFY
KIASSQARQVKLDPIRMLGFLLLVQCRRRVPSGLVTDQPSSRSAGSSPSLHAIWQIMQYYKVQGTYYAP
ALVVPSGAIWWIPPPGKRNIVSVQYLLTDLINLAILAHMTDMSPQLELAGVLMYLRAASSHSYAYTLL
QMKGVFPALSLRSMYRNKGFGGKAPAIEWTEPRSKYKFKWTGVTHLHDGLRPRSPAMDDATIATLSK
YELVDIGHMVINIRNQHPQHNHDSVRFVRDTMALTSGMYLVRQPTMSVLREYSQVPDIKEPIPPSAWT
GPIGNVRYLLPSVQGPARHLYDTWRAAARQIAQDPQWHDPLNQAIMRAQYVTARGGSSASLKFALK
VTGIVLPEYDDSKVKKSSKIYQAAQIARIAFMLLIAAIHAEVTMGIRNQVQRRARSIMPLNVIQQAISAP
HTLVANYINKHMNLSTTSGSVVTDKVIPLILYASTPPSTVVNVDIKACDASITYNYFLSVICGAMHEGFE
VGNADAAFMGVPSTIVSDRRSPVAPYSRPISGLQTMVQHLADLYAAGFRYSVSDAFSSGNKFSFPTSTF
PSGSTATSTEHTANNSTMMEYFLTVHAPSHVTSPSLKRILSDMTIQRNYVCQGDDGILLLPHEAASKVS
AEDMTELLDCLSQYGQLFGWKYDIDWSDTAEYLKLYALMGCRIPNTSRHPPVGKEYAAPQTDEIWPS
LIDIVIGHHLNGVTDVLNWREWLRFSWAFACYSSRGGYTNPRGQSFSAQYPWWTFVYLGIPPILLPGQ
TPFIHSCYMPPGDQGMFSILNGWRDWLIAHASSTLPPLRHPHPVWGLANVPSALTQFGVYAGYHAA
QHYRRPKPAPETASSDSISQITSDLTEYLFYDSALKARVMKGRYNWDRISTSLSLNVGSRVPSLFDVPGK
WVAAGRDAEKPPPSSVEEMFTSLNRCIRRPAQAFSRLLELYLRVHVALGESIPLAIDPDVPQVAGADPA
NDDHWFKYTCLGDIPSATRNYFGESLFVGRVVSGLDVEAVDATLLRLKILGAPPEAFIAVLNGIGMSDS
EAHQIAGRISLANAQLVQIARVVHLSIPSSWMTLNTGPYIHHHAYDFKPGLTQPSAKSRDKSVWMSPI
LKLLCTSYAMTVAGPVRTSIVTEIDGSAAALSGNLRVWMRDV
>S3
MPRRSARKAQSATNTSADTNVVPATDAPTSNSPPTTISPTQAAADANQQHAGISTSQSGPNAVGDSA
PSTSVNNDGDIITRPTSDSIASVANATKPAAVVSDQQAMKVTPIVNPSSYVCNVCNARFSTMSALSEHL
RSDHRDDASTLLATPMINNAIRSFLTAWDGLRILAPDISSKALSAYLDSAVANGPELIIEDTGLCTSFMLL
DNIPSAHLTEELIGFTWFMQMYQMTPPLPEGAVNRIVCMTNWASLGDEGRGLEVRLPPPTDSSVHAY
KTVLSRGYIDNVQFNPLALRSNVLLMLLQFTLSNLKINKSSTFTSDVTTLTSGRMIRAFEGRTELLALAY
PGRAVLPTQTKNAQFLSTAIPDRIGRLDRANLIGGEVSAMVECMELCDALTLNIRETYVMLLRSMHQD
PTQIVQIVNECANNLLNSTIPISLRPTILCPWFASSEDLRLQQVMHLVNISSNTAAALPLVEALSTLLRSV
TPLVLDPTVLTNAITTISESTTQTISPISEILRLLQPMGNDYAAFWKCIASWAYNGLVTTVLSEEAFPDSSQ

SITHLPSMWKCLFLTLAGPMTSDPHSPVKVFMALANLLAQPEPIAIGVPGMHQTTPASQFSHPGVWP
PGFLNPQLINPQQAPLLRAFAEHIRANWPQPSEFGYGSTLQGSANLFIPPNRMVYPWPNQPLPRLTVA
PTYDSAMSNWISTTVAFFIRVVNSVNMTATVNDLTRRTMTGVMTAMRQVKTMTPFYIQHMCPTELSV
LASVTVTPPFQVPFTRLVQNDVITNVLVARVDPAQRGDAAVDIRATHATFAAALPVDPASIVVAMLCG
QTETNLIPSHHYGKAFAPLFASNAMFTRNQRAVITREAFVCARSAVAQCQDAGFLVPRPLDALRQFDV
TSAAAAEIMHAVNDAFKTAFDLDGALLDGLALYGDPRIADLSAAYLQYGGNVVREHVPPGTSHIHR
ALQQVESTFMAEMNLFNVARGDLYLVQTATNGNWSPMAPVAAPPFVRGGPNVRVVGRFGTIVPRPN
GLEPQLIDDANVPQDIAGDWVYPSDVLQVSVAVFRDYVWPMVKAGRTRVLIELGHYVYTLHYYDPQI
SLDEAPILEEWLTHINPAGIPPVPFCIPTPQVYPCITARRVHYAFTSENNNDSLFSTNAASIDTAFGENAA
VSPLRWPGLVDPNYRVGTNDLPNRITLYNSLYRYNFTYPTLDGIMYVRSAT

>S4

MARRITLNSLKPLSAMNSHPSLTPTTTPTPATASTSAAPSNDSPATASLPAAFNQLVFEFNGVKYTGSPH
SWMMPYRGFEKHMVNMVLDTRPDATFSRFDDLLSAIVSALAVNGVTATTPFNEGELILLRFACLKTLR
ASAAAPTPSISDWSDSTPGPLLDSLDYGLPLSPSCEAAPPVLEPLRHSPLPPLHPDPAVSHSLVPMDVPD
SVLVPPSPTPATGAVIPSLLSLTLSTPPRSRASTASSSTPAVPRLSAAATPRRPTHIADDETYNRSRAAYA
MGVPALSPMYCTGKERHFEQTFYSAYPHAANGVWTAYQHSIILIAAPTEDISLLTLHHVEREQSATEAY
HLRALDGTNVARVAVCRLDPSMTCRDVHAMMAGHNVVSISGLAAAFMIRHRLTDGVFSKAFRRIVM
GIDPVMMRHDPVPLHLFAILTDHRLDHAATHAVMSMRIALMQVESASYQATKTWLRGHLPVTIFASA
TTDSSSDSIHATILEADKGMRTADTPGSSNLRELEASNAALQRQVIDMDVQVNALLRTISDLKSYTNH
QQASQGQTIQQYLHSHVCVNTKELAVLQSVMGEHAANAIQAVRVHANDTAKAALSEKVSVPLTAQL
SDTLAHLHAARSRNDELVAQLTIAESEAMAAAAERDQACELASELQTQVAAMQQEYENVTRALMQ
DNEQLQHSAATAEAAATSTFRPTPPLVYGTAPSALPAVGPGLTIPLLAPEIDPASLFL

>S5

MITIVVIPTAHFSWTDTNFLNSVDYRLTTQPKTRDRFAVYAPGWLRRQLDEFSASLTASELLQALESIPIP
VKARCLLLPKPKRFAQWLLDVPAANLWHIPVTTLRATVASKHPSSDIFNFIADHVPPNAEFDTITRRVP
AGKDIYVRTTKVIGAPLCLAAPAKYYAGYLSSHQLDSIYPDGWSPETFRKREYCLTILPSLLGPRTFLLD
VDAERDTTYPLSVLWPQLRAAALKSRLLLPPVALLRRVVDPSLKPIWSADTDPAFRALRLSRPSTASRP
AGFDFSALPIADLICLFESEPDSHGRTAPGTRLTLHAVPTDLLTALSIQENVRYPLRQQSGMFVHWVLL
ALLMSDDVSVTGTRRSIKMETAHASAKPFVHITIERCVSARLIDVRGSTAMYANAVCLTLPKGSYKSTII
DTLPAMFPDLPILDSNAVIDSDALGDSLRPSFELQFLERLNVLDAGVIDRAVASILNPTSDTSDDAAAM
VLETFNSLYRDVMTPAQRTRLPFLTQQGRVLAFAHSDYELLAANIPIQVIRGAIPIDHVVNLLARRNRV
GGTALQVLLDYCYRTQASPLATQPAGRLYKQLFGPWLMVPRLDTPLITLRLTATAPAKVLRAAGWTV
DGDPPLSVSCLRAYVTDRESAASLIEARLDSRALVTIDGDQIMFVEYAPPLPLVSIPRTFLLPVTYVAHW
VEPERVLLHGGNVSFTSGLEWTFADDPQGVTPAGV

>S6

MGNVQTSVNTYNINGDGNSFAPTSDMTSTAAPAIDLKPGVLNPTGKLWRPVGTTVATVDSLAIVSDRF
GQYSFVNENMRDTFAKALFDINMWQPLFQATKTGCGPIVLSAFTSTTSGYVGATAGDALDNFVTNGT
FISSVQILNLQRTIAARMRDVAHWQKHLDTAMTMLTPDISAGTASCNWKSLLAFAKDILPLDNLCLTY
PNEFYNVAVSRYPALRPGNVETKLPDAQSHPLGEVAGAFNAATSEVGSLVGSSSTLSQAISTMAGKDL
DLIEADTPLPVSVFTPSLAPRSYRPAFIKPEDAKWIAEFNNPAVIRQTLSYAGDDYTIQLGPGPTRVIDM
NSMIDAVLTLDVSGTPLPFQTNADLSSAIPAFVLIQTSVPIQQVTSASNITAVTVISAAEASAINLSVNVR
GDPRFNMLHLQATFERETIAGIPYIYGLGTFLIASITTSSDFCNPTLMDGLLTVTPVLLRETTYKGEVVDA
IVPATVMANQTSEEVASALANDAIVLVSNHLTKLANVVGDAIPVASKTDDSATSAIVSRLAVQHKLSQ
VGRTNPTPPDYPLLWRRAKRAASMFVSNPALALQVGIPVLTQSGMLSALTSGVGTALRTGSLGKGVT

DASEKLRARQSLTVAKQTFFDQIGSLWPGK
>S7
MNTNRSLIDERYDWIVAYERHIEPEAITAIRTPAYGALPPTQIPKFLGPNPKYFVYDGNVEEVEGQLIKD
PSDDYQPRRYADTWTCSLFTEVPPSHPSWPRDTVVQYDSTWSPYHHVPMRYFPRFMGLEDDLIKPND
GFWPILHRCAGFRFRYPWRFDLDALAAGIGTTVDNIRCIAESGAFATVVGTLRAMGWKYHHLSGLRP
QHVEYCVSAWALSFTREQLDDYKPGAWFDDLLRVPIYPDEQPIGLNREQLFTHPFVLLSWIRIQLLGGG
G
MPCQDTVSLSIQHTSVYVQHSCCVSTTTSASTSATALGLGCIACGIIGVIVVASGLCCLVNGRCSYCRGL
ALRSSSWKSPTTSLRTHQPISFGLRHLIGTDLRRPTHSRSLELLSDIHPVVPNRECPPPYDSLTVEPEVHTP
QTLQPQPRLQPPRW
>S8
MAQRQFFGLTYNFYGQPAPLYDLNDLQEIAGCYARPWTSRFSHLAISTGSLPVWSARYPSVAARNIIIN
TILGAHLNPFSGGQITSHQGITWRDPVLSSLVPVPAVQPPPAWAVAENVALDSNNYPTYVLNHSAMW
PVNQDVHVMTMWALSDQGPIYRLEVPAAPMPAATTAALMAYIGVPLAHLAQTAYKFAGQLPQSPDS
TMVSTIRWLSAIWFGSLTGRLNRSRTCNGFYFEFAKPSLNPDQAILKWNDGARVAAPAAAQVSYMRCI
SPHWQHQITEVAGAIMSQSVTAVTGLATLIDEATLPAWSQGVANLTGNGAGVTPCLDYNPVPMAAA
RHLQWRQDGLITAAQEAQMNADYTAYALTIEQHLTAMLAANPVAGGRMPIQPFVAADFGQAGQTA
AAVALAQAMFV
>S9
MAHTGTATLINAERTDRTLRLLETFPSLTITVRQDTSGRDLISSTYSATGMSAAARNLNPIANVKNMRQ
PGYVKPAHTPTNTVPLRVATSTGLELPTAHLAQMPVDQALRDAVAAAVPTHAAHVLPPQVDRVTPLT
LAARVAMVCAGLDCDDIHEIPPVPTAMALAFTTKVLLIHVVVDGDRASIAVNPDPARALLHADRLGN
IITNYGYDVRGTVRRDAAAALAPSELPAAYPIEWLGLICGLIATQIELDLDMLAMNQTEQKLVAPHVQ
AVDPFINRLQSYATLSARIMHLCVTHSQRPFRDFPELLRHWQKPELTTAIPINITLRGAALEVSGNGAEL
FRVRALPIGGM
>S10
MPLHMIPQVAHAMVRAATTGRLTMYTQTRTETTNYDHADYITCGRYTVCAYCLTTIAPHANVQTLPS
DHFCPRKPNEIAATLIEVSDKTRAALARSRTIDYHTLAVQAGFVEPTDDDDSSISGDITELPFRTCDLDV
ASASACVRSHCQAGHDGIHSLPIMTGDFKLPNERVHRAWGTGQSQNKVLTRCPTTGLLLAHDTQLR
ATSVVATAATRAVLMHSLLTSANASDGPQTRAACYGPSFSNLPYACHSACSADMAHFDCGYIVGLDL
HVEPSD
>S11
MATAGVTNAIDGLILSRLDEAITNNPTILRLRKKVSSLWKAYDNHVADPTAHELLTMPADLAELPPPLP
PTAEELNCSYYATAPPTPPPAVDTLWGTASYEIIGRETRLASSEKAPRIIIHVTDETIRLEVPTMAFTLPSAL
ATGSLLRVTLPKGAIHSALTGKFDERCWIRLTATLTKWMSPSTVVTSDWTLETPTGLIKPPVTGFNARA
TLRDSSLVLWFPAPNTSMIARRAIVSPMSCVFE
